# MetallopeptiGELs: Injectable Short-Peptide Supramolecular Hydrogels Self-Assembled by Pd Nanosheets for Bioorthogonal Therapy

**DOI:** 10.1021/jacs.6c19546

**Published:** 2026-10-22

**Authors:** Rafael Contreras-Montoya, Maria Sancho-Albero, Catherine Adam, Federico Movilla, Mari C. Mañas-Torres, Modesto T. López-López, Angel Orte, Victor Sebastian, Luis Álvarez de Cienfuegos, Asier Unciti-Broceta

**Affiliations:** † Cancer Research UK Scotland Centre, Institute of Genetics and Cancer, 214220 University of Edinburgh, Crewe Road South, EH4 2XU Edinburgh, U.K.; ‡ Nanoscopy-UGR Laboratory, Faculty of Pharmacy, University of Granada, E-18011 Granada, Spain; § Unidad de Excelencia Química Aplicada a Biomedicina y Medioambiente (UEQ), Universidad de Granada, E-18071 Granada, Spain; ∥ Instituto de Nanociencia y Materiales de Aragon (INMA), CSIC−University of Zaragoza, E-50009 Zaragoza, Spain; ⊥ Department of Chemical and Environmental Engineering Universidad de Zaragoza Campus Rio Ebro, E-50018 Zaragoza, Spain; # Networking Research Center on Bioengineering, Biomaterials and Nanomedicine (CIBER-BBN), E-28029 Madrid, Spain; ∇ Instituto de Investigación Sanitaria (IIS) de Aragón, Avenida San Juan Bosco, 13, 50009 Zaragoza, Spain; ○ POLYMAT and Department of Applied Chemistry, Faculty of Chemistry, University of the Basque Country UPV/EHU, Paseo Manuel Lardizabal 3, E-20018 Donostia-San Sebastián, Spain; ◆ Department of Applied Physics and Research Unit “Modeling Nature” (MNat), University of Granada, Avenida de Fuentenueva, E-18071 Granada, Spain; ¶ Instituto de Investigación Biosanitaria ibs.GRANADA, Av. de Madrid, 15, E-18016 Granada, Spain; †† Laboratorio de Microscopías Avanzadas, 16765 Universidad de Zaragoza, E-50018 Zaragoza, Spain; ‡‡ Department of Organic Chemistry, University of Granada, Avenida de Fuentenueva, E-18071 Granada, Spain

## Abstract

Controlling the delivery of chemotherapy to the tumor site is a long-standing goal of the medical field, as it could not only enhance the anticancer activity of the treatment but also reduce damage to the rest of the body. The emergence of bioorthogonal catalysis as a strategy capable of mediating continuous drug production in a nonenzymatic fashion has created the opportunity to develop so-called “bioorthogonal therapies,” which aim to generate the anticancer agents inside the tumors. Herein, we describe a novel class of supramolecular materials comprising ultrathin palladium nanosheets and fluorenylmethoxycarbonyl-diphenylalanine that self-assemble in water to form injectable, self-healing, biocompatible catalytic gels that convert inactive prodyes and prodrugs into active small molecules. The relatively weak physicochemical cross-links generated by the hybrid metal-peptide fibers facilitate the flow of the gel under mild shear stress (shear-thinning), thereby enabling precise intratumoral injections *in vivo* and localized prodrug activation. The strategy was validated in an aggressive pancreatic cancer xenograft model in mice, setting out a new direction for the field of bioorthogonal therapies.

## Graphical abstract

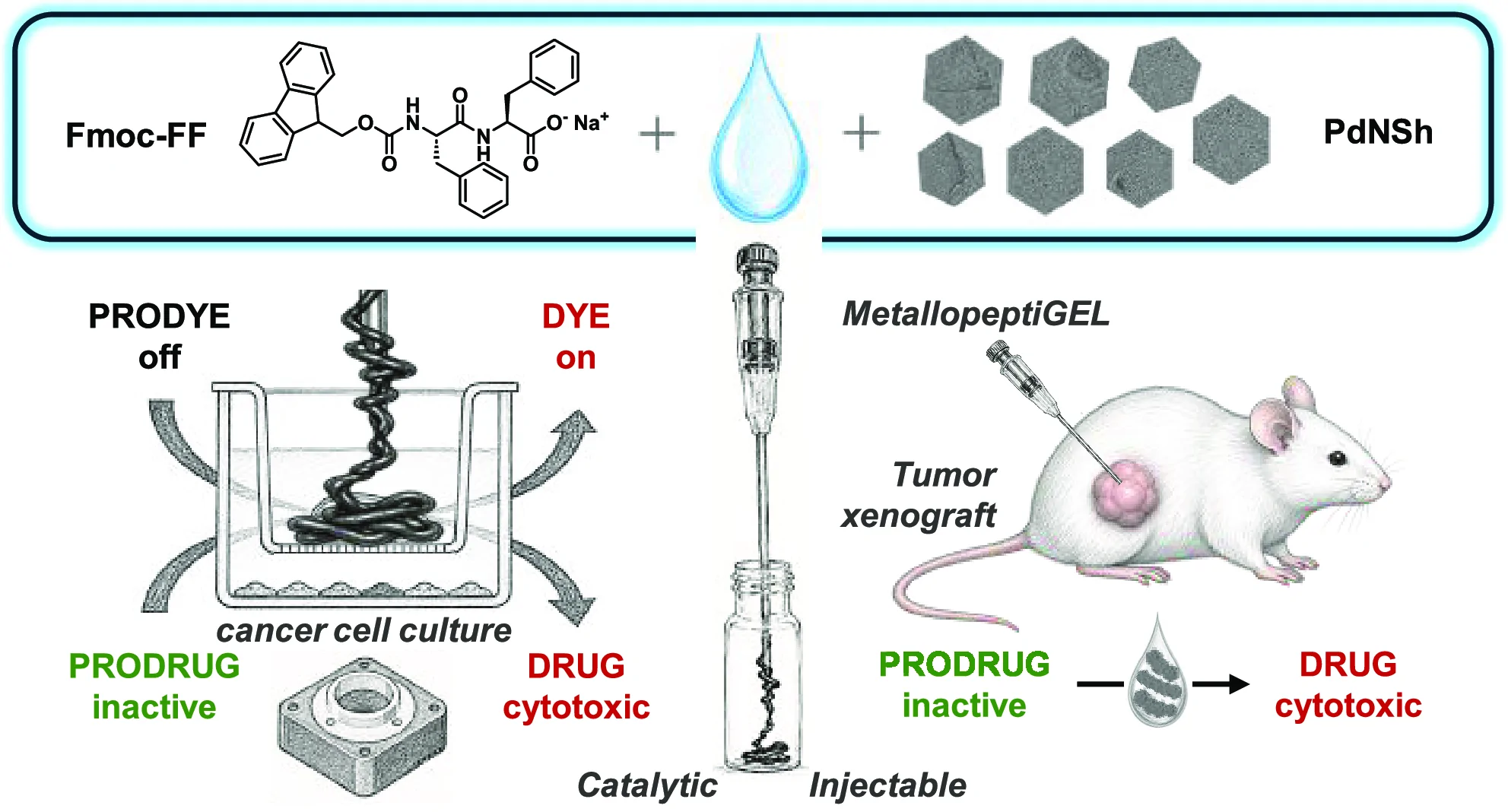

## Introduction

Bioorthogonal catalysts are metal-based tools designed to produce supramolecular quantities of functional molecules in living systems through bioindependent chemical processes.^1–4^ The main motivation behind these strategies is to control where and when such reactions take place, which creates the opportunity to manufacture or uncage bioactive molecules at precise anatomical areas. This goal has inspired researchers to develop multiple strategies to incorporate abiotic transition-metal catalysts into a wide variety of formulations, including organometallic complexes, MOFs, nanozymes, polymeric materials, biological vesicles, and immune cells.^5–32^

Among these approaches, a small number of studies have explored the use of natural (alginate) and nonbiodegradable gelling agents (poly­(2-hydroxyethyl methacrylate)) to immobilize metal nanoparticles into a semisolid 3D network.^33,34^ This aims to enable small-molecule diffusion through the water-rich network to facilitate interactions between substrate molecules and catalyst active sites while maintaining the catalytic metals trapped into a biocompatible structure. The ultimate purpose of this approach is to provide an off-the-shelf injectable gel-embedded catalyst that can be implanted at the desired anatomical location, *e.g.*, a tumor, to mediate the uncaging and activation of anticancer drugs that have been systemically administered as inactive precursors. However, previous proof-of-concept studies^33,34^ could not progress to animal studies because of the poor mechanical properties and limited catalytic activity and durability of the gels.

In an ideal scenario, the gel-metal mixture should feature mechanical properties such that it could facilitate intratumoral injection, diffuse and spread throughout the tumor, while, at the same time, remaining at the malignant tissue to minimize loss of the metallic catalytic cargo. Therefore, ideal metallogels for this purpose must flow under stress (shear-thinning), recover their original stiffness after stress cancellation (self-healing), and remain catalytically active in the chemically heterogeneous environment of the tumor. Such a kind of material, which to the best of our knowledge has not yet been reported, could facilitate a minimally invasive medical procedure for surgeons to deliver catalysts into tumors. The bioorthogonal therapy strategy would be fully realized by the subsequent systemic administration of the inactive prodrug, which would be activated exclusively where the catalyst was located. Such an approach would treat the cancer *glocally* (*globally* administered prodrug, *locally* released drug) and enable repetitive prodrug dosing due to the catalytic nature of the drug activation process.

Some types of self-assembling peptides^35–39^ (*e.g.*, β-hairpin peptides) and aromatic short peptides^40,41^ have been shown to form shear-thinning hydrogels. Aviv et al. reported that fluorenylmethoxycarbonyl-diphenylalanine (Fmoc-FF) in combination with hyaluronic acid at specific ratios can display shear-thinning properties,^42^ and we showed that Fmoc-FF in combination with Fmoc-arginine-glycine-aspartic acid (Fmoc-RGD) and iron nanoparticles improved the injectability properties of the metallogel.^43^ Nevertheless, in both cases, peptide self-assembly was triggered by modifying media conditions, which is not optimal for therapeutic use as the gel could be susceptible to chemical variations across the tumor tissue (*e.g.*, changes in pH, oxygen, and nutrient levels depending on the distance to capillaries). Therefore, we wondered if this type of peptide could self-assemble by an abiotic stimulus that cannot be affected by biological environments; specifically, we speculated if these peptides could form gels triggered by the addition of a metallic catalyst. To explore this concept, we investigated the use of ultrathin palladium (Pd) nanosheets (PdNSh),^44,45^ which are 2D nanostructures, highly catalytic and capable of mediating bioorthogonal depropargylation reactions.^23,33,34^ Our hypothesis consisted of the postulation that aromatic Fmoc-dipeptides could interact with planar metallic Pd nanosheets through π–metal interactions^46,47^ and self-organize into metal-peptide fibers with the capacity to form supramolecular gels, enable small-molecule substrate diffusion, catalyze bioorthogonal reactions and, at the same time, facilitate *in vivo* injections with simple surgical materials.

Here we show that Fmoc-FF can form a homogeneous hydrogel in the presence of PdNSh, resulting in a soft composite material that features shear-thinning and self-healing properties. Computational studies indicate that Fmoc-FF has a strong tendency to adhere on the Pd nanosheets’ surface, preferably interacting through its aromatic moieties. These aggregates can then act as anchoring centers for peptide supramolecular polymers, favoring gel formation. Notably, hydrogel-embedded PdNSh effectively mediate depropargylation reactions in serum-containing media, with superior reactivity to freestanding PdNSh. We also show that this metallohydrogel, tentatively named metallopeptiGELs, can mediate the uncaging of a chemotherapeutic drug in cancer cell culture. The therapeutic potential of this first-in-class injectable catalytic gel was validated in a pancreatic cancer xenograft model in mice, demonstrating strong anticancer effect only in combination with a bioorthogonally activated anticancer prodrug.

## Results and Discussion

### Design, Preparation, and Mechanical Characterization of MetallopeptiGELs

Supramolecular polymers based on Fmoc-dipeptides, particularly Fmoc-FF, have been an area of intensive research for the last 20 years.^48–54^ The mechanism of self-assembly usually follows a nucleation-elongation process, which also implies the formation and evolution of different polymorphs through an Oswald ripening process.^55^ The resulting long fibers formed by self-assembly are then able to intertwine, giving rise to a 3D network able to macroscopically immobilize the water media. Different strategies have been developed to promote gel formation, *i.e.*, to increase the formation of the most thermodynamically stable fibers. For instance, the so-called “solvent switch” strategy involves the addition of a highly concentrated solution of Fmoc-FF in an organic solvent (usually in DMSO) into a high volume of water, in which this peptide is not soluble, thus triggering peptide aggregation. Another strategy named “pH switch” initiates by solubilizing Fmoc-FF in water by adding a base (usually NaOH) to form the Fmoc-FF Na^+^ salt. Gel formation is then carried out by carefully lowering the pH through acid addition. The neutralization process regenerates the neutral insoluble form of Fmoc-FF, inducing aggregation. Chen et al. showed that the self-assembly of the Fmoc-FF Na^+^ salt can also be promoted by the addition of different metallic salts,^56^ whereas we demonstrated that the nature of the metallic cation has a strong impact on the mechanism of self-assembly and on the resulting physical properties of the gel.^57^ Interestingly, these being interactions of ionic nature, gels formed by the addition of metallic salts usually show self-healing. Gazit et al. showed that the nature of the metallic cation can also affect the secondary structure of the fibers from the more frequent β-sheets to other conformations.^58^

Although none of these methods were designed to withstand the chemically evolving tumor microenvironment, the current understanding of Fmoc-FF self-assembly and gel formation inspired us to investigate π–metallic interactions between aromatic rings and metal surfaces as a nucleation initiator. These types of interactions are strong at ambient conditions, particularly for metals of the VIIIB group, and are known to be responsible for relevant industrial processes.^47^ Based on our expertise with PdNSh,^23,44,45,59^ we performed a series of formulation studies to investigate whether these ultrathin metallic structures (<2 nm in thickness, Figures 1a and S1) could trigger the formation of Fmoc-FF-based hydrogels. To our delight, mixing an Fmoc-FF alkaline solution with a colloidal suspension of PdNSh (protocol described in the experimental part in the Supporting Information) instantly formed a soft gel (Figure 1b). Further homogenization by vortexing and sonication yielded a homogeneous black metallopeptiGEL that, due to its fluidic nature, facilitated being loaded into a syringe and subsequent extrusion through its needle into a water solution without losing its integrity (Figure 1c and Supporting Movie 1). This experiment not only proved the shear-thinning properties and injectability of the gel but also its self-healing capacity.

**Figure 1:**
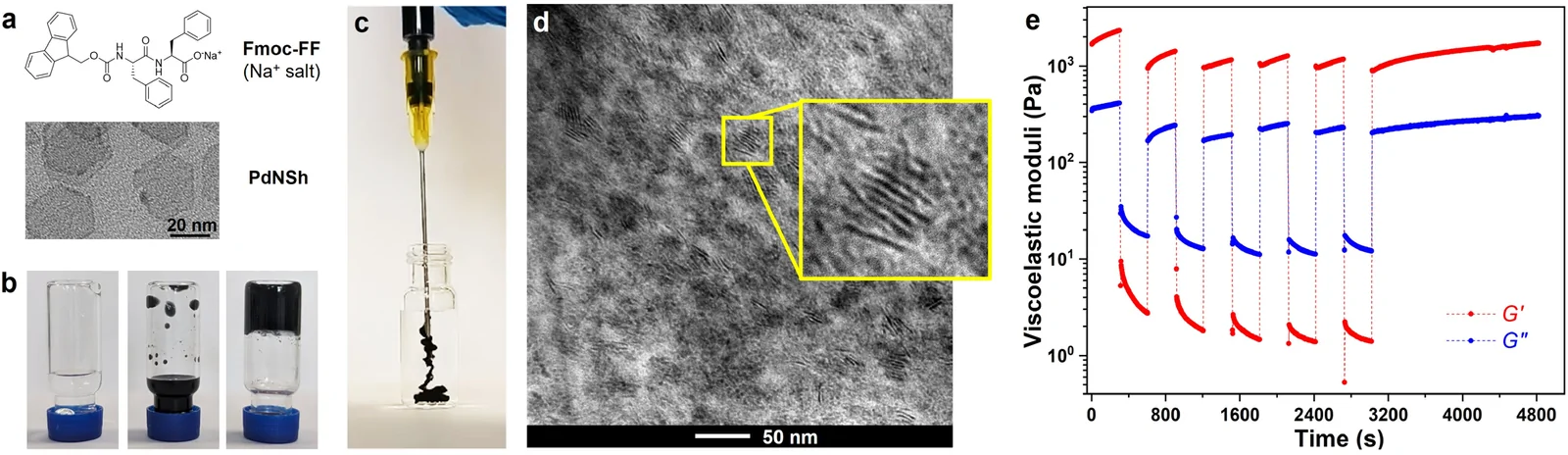
(a) Structure of Fmoc-FF and image of PdNSh. (b) Inverted-tube test of Fmoc-FF alkaline solution (left), PdNSh aqueous suspension (middle), and metallopeptiGEL (right). (c) Image of the extrusion of preformed metallopeptiGEL through a syringe into a vial with water. (d) TEM micrograph revealing the directional alignment of PdNSh within the gel matrix. The PdNSh are predominantly observed in the edge-on orientation, appearing as thin nanostructures along a preferential direction. Inset: Higher magnification view highlighting the detail of individual PdNSh to appreciate their uniform thickness. (e) Self-healing behavior evaluated by time sweep oscillatory rheology at 1 Hz; samples were subjected to five cycles alternating between low (γ_0_ = 0.001) and high (γ_0_ = 1.5) strain amplitudes, followed by a final recovery step at γ_0_ = 0.001.

Next, transmission electron microscopy (TEM) of freshly prepared metallopeptiGELs was performed (Figures 1d and S1). Notably, the images reveal that the PdNSh is primarily transversally oriented within the hydrogel network. Consequently, instead of the characteristic hexagonal geometry observed in the isolated colloid (Figure S1), the edge-on view of the nanosheets is predominantly visible. This orientation allows for the direct observation of the nanosheet thickness as they are integrated and intercalated between the short Fmoc-FF peptide pseudofibers.

To evaluate the mechanical properties of the metallopeptiGELs, including their self-healing, rheology studies under shear stress were performed next. The mechanical behavior was evaluated by keeping materials at 37 °C to emulate physiological conditions. Test results under oscillatory shear at a constant frequency of 1 Hz and increasing strain amplitude (amplitude sweep) revealed a behavior typical of a weak gel, characterized by values of the storage modulus (*G′*) less than 1 order of magnitude larger than those of the loss modulus (*G*″) at low strain amplitude (Figure S2a). This region of low strain amplitude is commonly identified as the linear viscoelastic region (LVR), for which a proportionality between amplitudes of stress and strain is expected, as well as values of *G′* and *G′*′ approximately independent of the strain amplitude. However, the metallopeptiGELs demonstrated a decrease of both *G′* and *G′*′ at very low values of strain amplitude, which is also an indication of the weak character of these hydrogels. At moderate-to-high strain amplitude, a crossover of *G′* and *G″* takes place because of the breakage of the hydrogel network and the consequent transition to a liquid-like state, for which *G′* < *G″*. To further characterize the mechanical properties of these materials, their response to oscillatory shear of constant amplitude $\gamma_{0}={5\cdot 10}^{-3}$ and increasing frequency (frequency sweep) was investigated (Figure S2b). The study provided larger values of *G′* than *G″*, confirming prior observations. Furthermore, the increase of these two moduli with frequency further indicated the mechanical weakness of the material, which can be categorized as weak gels.

Finally, the self-healing potential of these materials was investigated by subjecting them to alternating pulses of low and high strain amplitude of oscillatory shear (1 Hz frequency) (Figure 1e). As observed, at low strain amplitude, a gel-like behavior (*G′* > *G″*) is observed, whereas at high strain amplitude, a liquid-like behavior (*G′* < *G″*) is seen. However, as the strain amplitude returned to a low value, the hydrogels switched back to a gel-like behavior (*G′* > *G″*). Although this occurred, the previous high values of *G′* and *G″* were not completely recovered within the time frame of the analyzed pulses. The hydrogels exhibited progressive weakening under successive pulses of high strain amplitude, evidenced by the gradual decrease of both *G′* and *G″* within and after these pulses. Although the remarkable self-healing behavior of the composite was demonstrated by a progressive enhancement of *G′* and *G″* at low strain amplitude, these quantities did not fully recover their previous low-strain values. This is noteworthy as it suggests that, despite the notable self-healing capacity of the material, it can adapt to imposed physical restriction, which would be optimal for implantation in tumoral tissue.

### Small-Angle X-ray Scattering and Molecular Dynamics Analysis

To shed light on the role of the PdNSh in the process of Fmoc-FF self-assembly, small-angle X-ray scattering (SAXS) experiments as well as molecular dynamics (DM) simulations were conducted. SAXS data were modeled using a cylindrical form factor, incorporating a surrounding hard-sphere potential to account for interparticle interactions (Figure 2a, inset). The necessity of including this hard-sphere potential to satisfactorily fit the SAXS data may indicate an interaction between the PdNSh in solution, inhibiting the free rotation of the particles.

**Figure 2:**
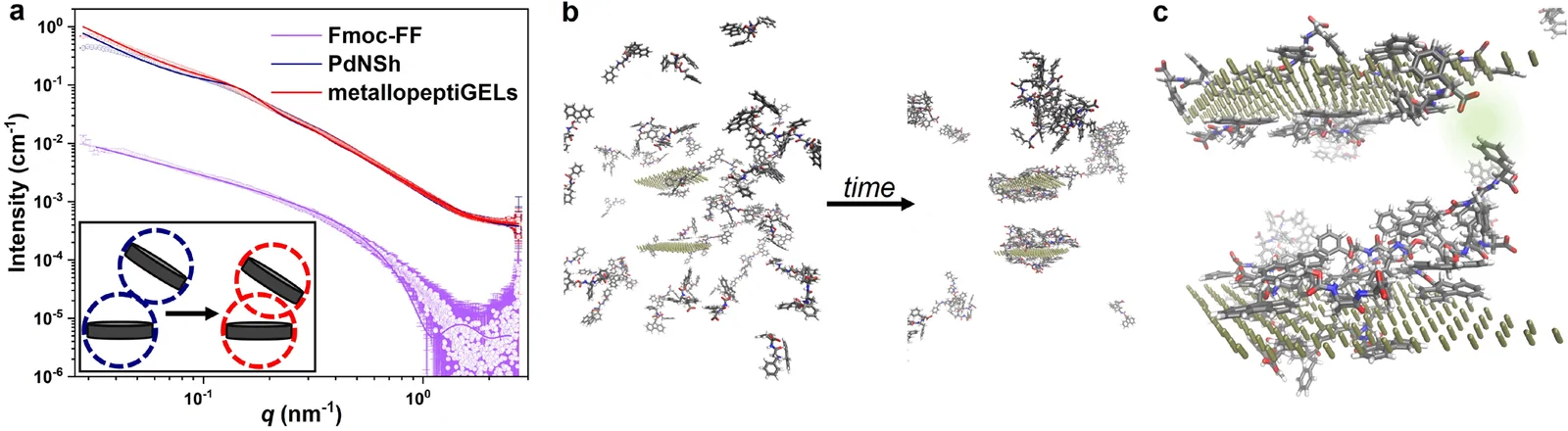
(a) SAXS profiles showing the scattering intensity *I*/*q* as a function of the scattering vector *q* for Fmoc-FF solution, PdNSh suspension, and metallopetiGELs. Solid lines represent fits obtained using a cylindrical form factor incorporating a surrounding hard-sphere potential. The inset schematically illustrates the fitting model for PdNSh evolving to metallopeptiGELs after gelation. (b) MD simulation of Fmoc-FF self-assembly in the presence of two Pd slabs simulating nanosheets showing the starting point (left side) and the end of simulation (right side). (c) Details of the MD simulation at the final point, highlighting intermolecular interactions.

Interestingly, when the same cylindrical-disk model with hard-sphere interactions was used to fit the metallopeptiGELs, it also provided satisfactory results (Figure 2a). However, in the case of the gel, both the effective radius and the occupied volume fraction changed: the cylindrical radius increased—likely due to Fmoc-FF molecules surrounding and adhering to the surface of the PdNSh—while the occupied volume fraction decreased, which indicates that the Pd nanosheets pack more tightly, thus occupying a smaller volume. Interestingly, the interacting hard-sphere model was also consistent with the results obtained from MD simulations (Figure 2b,c), which clearly show how Fmoc-FF molecules adhere to the Pd surface, fully surrounding the particle in a manner reminiscent of the organic shell in certain core–shell systems. Additionally, the simulations revealed that Fmoc-FF molecules may occupy the internanosheet space by forming supramolecular chains that extend from both surfaces (Figure 2c). The sum of these calculations suggests that PdNSh covered with Fmoc-FF molecules might act as anchoring sites for supramolecular peptide polymers, promoting self-assembly and working as dynamic network nodes. In turn, these interactions may also favor the self-healing process after gel disruption. Notably, the analyses suggest that all of these supramolecular modifications—both at the surface and in the interparticle regions—occur without disrupting the classical supramolecular arrangements typically formed by Fmoc-FF in solution. The formation of these interparticle supramolecular “sticky” chains not only increases and extends long-range interactions between particles—facilitating the formation of a spanning network that entraps the liquid phase and imparts the rheological properties of a gel—but may also function analogously to ropes anchoring a floating boat. These interactions may help PdNSh to align in a face-to-face fashion, as it is observed in the TEM images of the metallopeptiGELs (Figures 1d and S1).

### Functional Characterization of MetallopeptiGELs *In Vitro* and in Cell Culture

A fluorogenic test was performed to evaluate the bioorthogonal catalytic capability of metallopeptiGELs versus naked PdNSh under physiological conditions. Small portions of gel were added to a 100 μM solution of the Pd-labile bioorthogonal prodye **Poc-eNBD** in PBS with or without serum to get a final concentration of 40 μg/mL Pd (Figure 3a). For comparison, naked PdNSh were dispersed in the reaction medium at the same concentration. Reactions were incubated at 37 °C, and changes in fluorescence intensity were measured with a plate reader (Ex/Em: 440/535 nm) after 1, 2, 4, 8, and 24 h. The analysis revealed that the metallopeptiGEL-mediated conversion of prodye into dye (**eNBD**) is enhanced in the presence of serum (Figure 3b; see results in PBS in Figure S3, Supporting Information), outperforming the activity of naked PdNSh. The catalytic activity and biocompatibility of the catalytic gel in cell culture were further confirmed by placing the gel (40 μg/mL Pd) in a transwell insert, incubating with BxPC-3 pancreatic cancer cells and monitoring the activation of a prodye (**Pro-Res** ^23^) in a microscope-in-incubator imaging platform (Incucyte system, Figure S4, Supporting Information). Notably, Pd leaching determination from the gel after 5 days of incubation in serum-supplemented media showed less than 5% loss of Pd content (measured by ICP-MS, see the Supporting Information). The leached Pd displayed negligible catalytic activity, indicating that free Pd species are rapidly deactivated in biological media.

**Figure 3:**
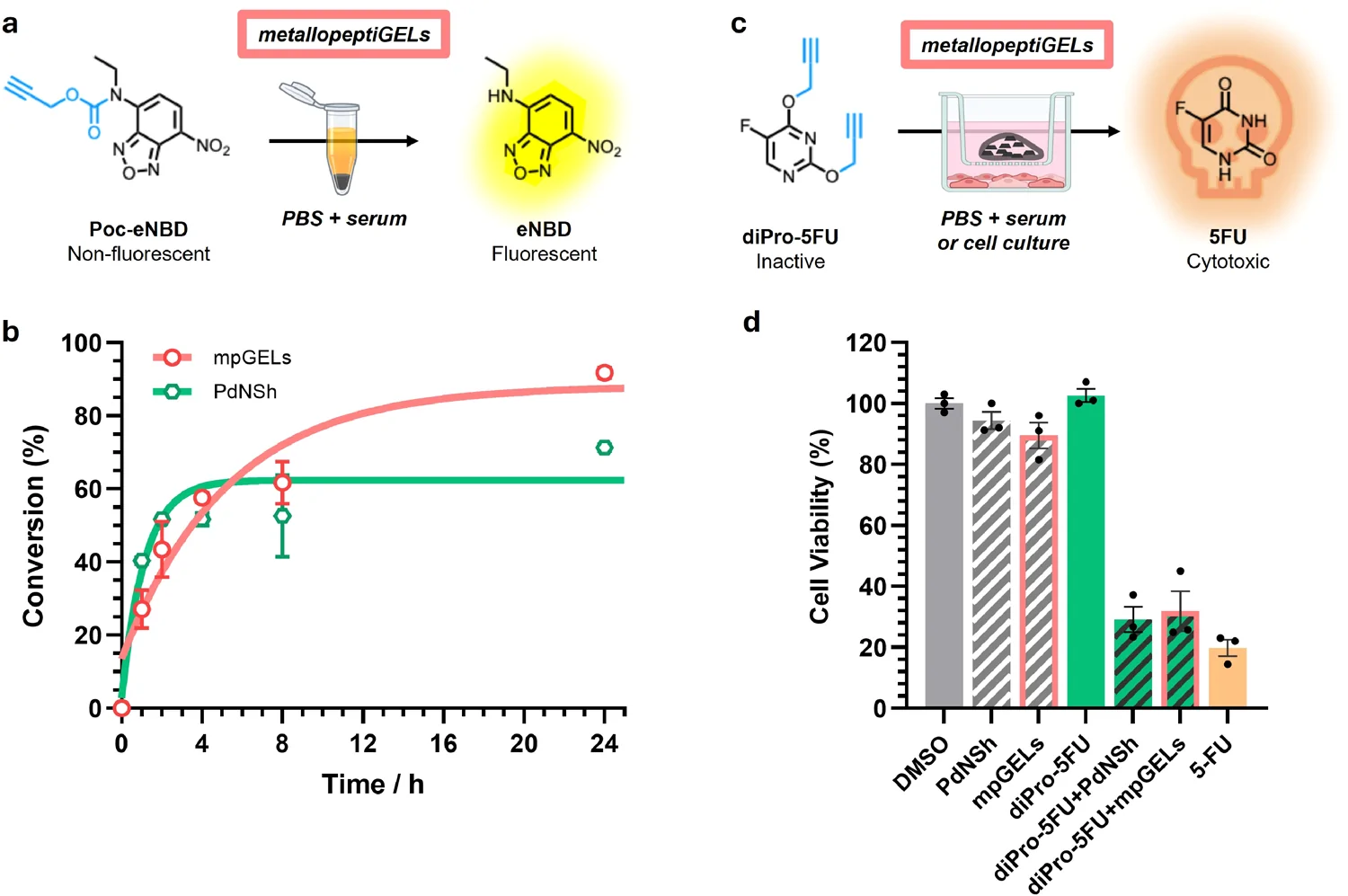
(a) Palladium-mediated uncaging of **Poc-eNBD** into fluorescent **eNBD**. (b) Kinetic profile of the uncaging of **Poc-eNBD** (100 μM) catalyzed by metallopeptiGELs or PdNSh (40 μg of Pd/mL) under physiological conditions (37 °C, PBS) in PBS with 10% serum. (c) Setup of the catalytic gel-mediated activation of **diPro-5FU** in cancer cell culture. (d) Cell viability of BxPC-3 pancreatic cancer cells after 5 days of treatment with **diPro-5FU** (100 μM) with or without metallopeptiGELs or PdNSh (29 μg of Pd/mL), compared with positive control drug 5FU (100 μM) alone. *n* = 3. Error bars = SEM.

### Prodrug Activation Studies

The pyrimidine analog **5FU** is an antimetabolite drug approved for pancreatic cancer treatment, one of the most aggressive forms of cancer.^60^ The capacity of metallopeptiGELs to function as a catalytic activator of chemotherapy under biocompatible conditions (37 °C, pH 7.2) was first studied *in vitro*, by incubating the caged derivative **diPro-5FU** (designed to release **5FU** after double Pd-mediated depropargylation^60^) with metallopeptiGELs in cell culture media. Direct HPLC analysis of the mixture verified **5FU** formation (Figure S5, Supporting Information), further confirming the reactivity of the catalytic gel in complex media. Next, the strategy was tested in BxPC-3 pancreatic cancer cell culture. Inactive prodrug **diPro-5FU** (100 μM) was incubated for 5 days with BxPC-3 cells alone or combined with metallopeptiGELs (29 μg/mL Pd) supported in a transwell insert. As expected, in the absence of a catalyst, the prodrug did not induce any toxic activity, and in the absence of the prodrug, the catalysts had minimal impact on cancer cell viability. In contrast, the combined prodrug and metallopeptiGELs treatment resulted in significant cell death, equivalent to the cytotoxicity observed for the combination of prodrug and naked PdNSh and slightly lower than the positive control drug **5FU** (Figure 3d).

To confirm that the cytotoxic mode of action of the bioorthogonal therapy approach (metallopeptiGEL + **diPro-5FU**) is analogous to that of **5FU**, an apoptosis assay was performed using a Caspase-3/7 off-on probe (CellEventTM Caspase-3/7) and monitored for 4 days with the Incucyte system. As shown in Figure S6 (Supporting Information), direct drug treatment led to equivalent levels of apoptosis as the combination of the catalytic gel and the prodrug, in agreement with the in situ generation of **5FU**.

### *In Vivo* Biodistribution Study

Encouraged by the properties of the metallopeptiGELs, a BxPC-3 pancreatic cancer xenograft in mice was employed to evaluate their *in vivo* potential as an implantable bioorthogonal catalyst. A Pd metal biodistribution experiment was first conducted to measure the amount of Pd retained in the tumor and its level of distribution to other organs after intratumoral injection of the Pd-loaded gel (see full description of the experiment in Figure S7, Supporting Information). 15 or 30 μL of metallopeptiGEL (1 μg of Pd/μL of gel) was readily injected into the tumors using an insulin syringe. The presence of Pd species was then quantified in tumor, kidneys, liver, lungs, spleen, and pancreas by inductively coupled plasma optical emission spectroscopy (ICP-OES) after organ digestion 1, 2, or 7 days after the injection (Figures 4a, S8, and S9). For both doses, significantly higher levels of metal were localized in the tumors, even one week postinjection, indicating that the metallopeptiGELs are successfully retained in the tumor. In contrast, small amounts or just traces of Pd were present in the liver, spleen, pancreas, lungs, and kidneys. When normalizing the amount of Pd by tumor weight, around 25 and 50 μg of metal per g of tumor were retained by the tumor, respectively. The minimal variation of intratumoral Pd content between 1 and 7 days may be explained by the expected retention of PdNSh within the tumor microenvironment due to their defective lymphatic drainage system,^61^ which results in an intrinsic barrier for these nanostructures to diffuse through the solid tumor matrix, access the bloodstream, and subsequently distribute to other organs. Figure S9a,b (Supporting Information) also includes Pd amount normalized by organ weight for animals treated with 30 and 15 μL, respectively. This normalization facilitates the comparison of Pd levels across organs by accounting for differences in tissue weight, further confirming the superior retention of the metal in the tumor. As qualitative studies, representative confocal microscopy and histology images are included in Figure S10 (Supporting Information) and Figure 4b, respectively, to visualize the presence of Pd in the treated tumors. Of note, histology analysis of tumors did not exhibit significant morphological alterations 1 week after injection of the metallopeptiGELs.

**Figure 4:**
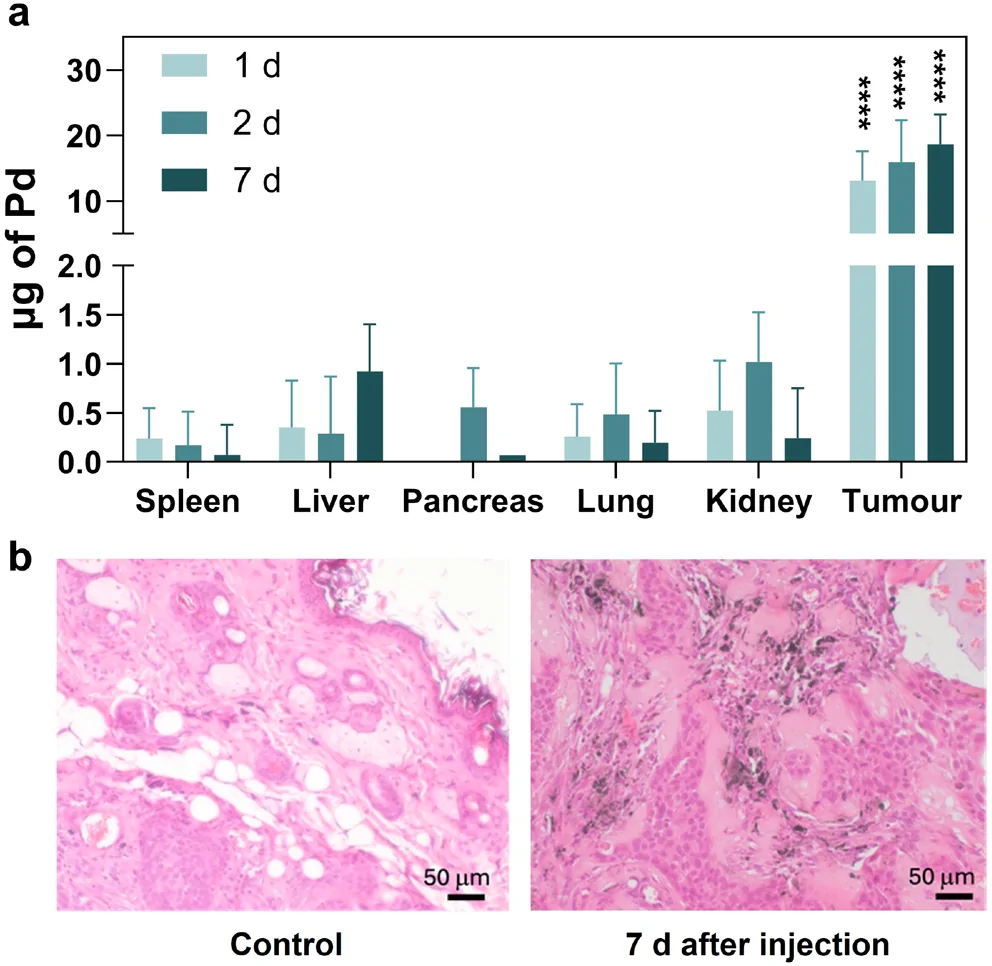
Pd biodistribution after intratumoral (IT) injection of metallopeptiGELs (30 μL). (a) ICP-OES analysis of Pd content in organs extracted from mice 1, 2, and 7 days after metallopeptiGEL administration. Results are expressed as mean ± SD. Statistically significant differences are expressed as ****p < 0.0001. (b) Representative histology images (hematoxylin-eosin staining) of tumors without treatment (control) or 7 days after IT injection with 30 μL of metallopeptiGELs. PdNSh of the metallopeptiGELs are visualized as dark spots within the tissue.

### *In Vivo* Efficacy Study

We next performed an efficacy study in the same xenograft model of pancreatic cancer to evaluate the local metallopeptiGEL-mediated bioorthogonal release of **5FU** from systemically administered **diPro-5FU**. Four arms (*n* = 11) were tested in the study: untreated control; **diPro-5FU** only (negative control); metallopeptiGELs only (negative control); and coadministration of metallopeptiGELs and **diPro-5FU** (see full description of the experiment in Figure S7, Supporting Information). After tumor formation, animals were subjected to two or three cycles of bioorthogonal therapy, consisting of metallopeptiGEL intratumoral (IT) injection (30 μL/day) followed by four consecutive days of **diPro-5FU** intraperitoneal (IP) administration (150 mg/kg). Each cycle was separated by 2 days without treatment. Tumor volumes were measured every 2 or 3 days with a caliper and mice were weighed to monitor toxic events. Notably, the weight of the animals remained similar between the different animal cohorts through the study (see Figure S11, Supporting Information), indicating that the treatments were well tolerated.

After two cycles of bioorthogonal therapy, mice of each group (*n* = 5) were randomly selected and sacrificed at day 20, and tumors were taken to analyze the level of apoptosis by Caspase-3 immunohistochemistry (see methods in the Supporting Information). Figure 5a shows the percentage of positive Caspase-3 cells, and Figure 5b displays representative images of tumors from each group. Both studies clearly showed significantly higher numbers of apoptotic cells in the tumors of mice treated with both metallopeptiGELs and **diPro-5FU**, indicating that the prodrug was locally converted into **5FU**.

**Figure 5:**
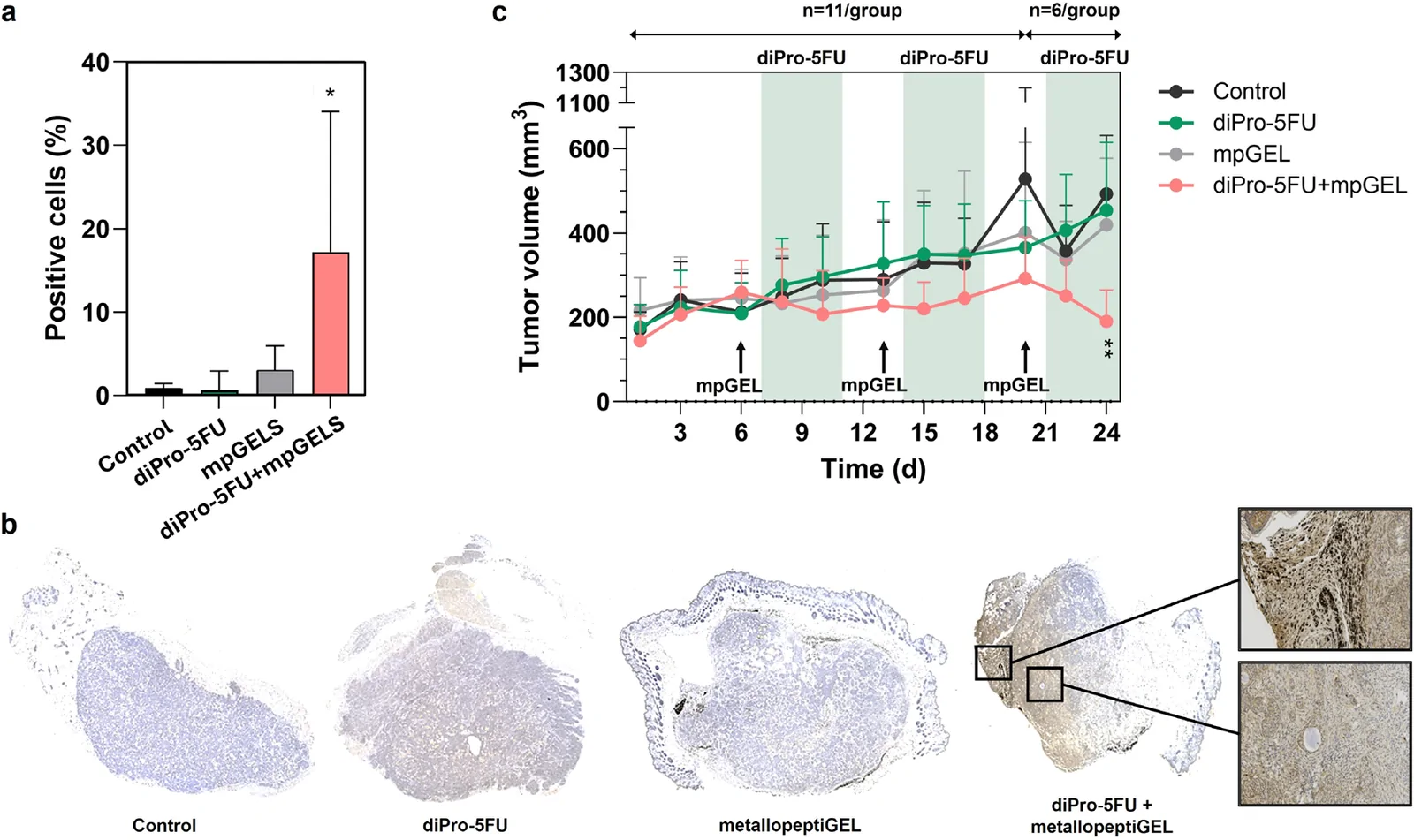
Bioorthogonal catalysis *in vivo* study of xenograft tumors treated with metallopeptiGELs and **diPro-5FU**. (a) Quantification of Caspase-3-expressing cells from xenograft samples of the different experimental groups after 2 cycles of biorthogonal therapy (*n* = 5/group). (b) Representative immunohistochemistry images of xenograft samples from the different experimental groups after Caspase-3 staining: control, **diPro-5FU** (150 mg/kg, IP), metallopeptiGELs (30 μL, IT); metallopeptiGELs (30 μL, IT) plus **diPro-5FU** (150 mg/kg, IP). (c) Tumor growth evolution of BxPC-3 xenograft-bearing mice over 2 (*n* = 11) or 3 cycles of treatment (*n* = 6). The days of metallopeptiGEL injections (mpGEL) are indicated with black arrows, and the four consecutive days of **diPro-5FU** administration are noted with a green background.

The mice remaining in the study (*n* = 6) were subjected to a third bioorthogonal therapy cycle. Overall, interval reduction of tumor volumes was observed during the length of the study in days that concurred with each bioorthogonal therapy cycle (see Figure 5c). The most prominent effect was observed after the third cycle, which led to significantly smaller tumor volumes than the control groups (*P* < 0.01). Measurement of tumor weights after sacrificing the mice (see Figure S12) confirmed this trend. The results of the *in vivo* studies prove the capacity of the PdNSh entrapped in the metallopeptiGELs to uncage systemically administered **diPro-5FU** at the tumor site to induce localized apoptosis and tumor shrinkage. This is noteworthy because catalytic devices limited by low distribution throughout the tumor (*e.g.*, Pd microdevices^60^) previously failed to affect overall tumor growth using the same prodrug.

## Conclusion

In our search to find novel functional materials with scope to progress to medical practice, we investigated the development of “catalytic gels” more suitable for storage, shipment, and clinical use than previously reported catalytic devices. Our research efforts successfully uncovered that the short-peptide Fmoc-FF forms a catalytically active gel (metallopeptiGELs) in water when exposed to ultrathin PdNSh. The resulting gel can be injected into tumors, where it adheres to malignant tissue and limits the spread of the metallic catalyst. Computational studies suggest that Fmoc-FF’s affinity for PdNSh promotes aggregate formation, aiding gelation. The implanted metallopeptiGELs remain catalytically active in tumors, enabling site-specific release of an approved chemotherapy drug and inducing cancer cell apoptosis. This bioorthogonal therapeutic strategy demonstrated anticancer efficacy in a mouse model of pancreatic cancer, one of the most aggressive forms of cancer, when both the catalyst and the drug precursor were administered. The injectable catalytic gel reported herein, which to the best of our knowledge is the first of its kind, offers a novel approach to use bioorthogonal strategies to treat disease. We believe this investigation will inspire researchers to further explore the therapeutic potential of bioorthogonal catalysis.

## Supplementary Material







## Notes

Homogenization by vortexing and sonication yielded a homogeneous black metallopeptiGEL that, due to its fluidic nature, facilitated being loaded into a syringe and subsequent extrusion through its needle into a water solution without losing its integrity (Movie 1) (MP4)
Timelapse of all timepoints (Movie 2) (MOV)
Detailed materials and methods, nanomaterials characterization data, compound synthesis and characterization, experimental procedures for biological and *in vivo* studies, references, and Figures S1−S12 (PDF)

## Abbreviations

5FU: 5-fluorouracil
FDA: 5-fluorouracil
NPsA: nanoparticles
HAADF: high-angle annular dark-field
(S)­TEM: (scanning) transmission electron microscopy
EDS: energy-dispersive X-ray spectroscopy
ICP-MS: inductively coupled plasma mass spectrometry
ICP-OES: inductively coupled plasma optical emission spectroscopy
HR-TEM: high-resolution transmission electron microscopy
