# Supplementary material for: MetallopeptiGELs: Injectable Short-Peptide Supramolecular Hydrogels Self-Assembled by Pd Nanosheets for Bioorthogonal Therapy
Source: J Am Chem Soc. 2026 Oct 22;148(43):47607–15. doi: 10.1021/jacs.6c19546 (PMC13741829; doi:10.1021/jacs.6c19546)
Supplement: Supplementary file 1 [file ja6c19546_si_003.pdf]

Supporting Information

MetallopeptiGELs: Injectable Short-Peptide Supramolecular Hydrogels Self-Assembled by Pd Nanosheets for Bioorthogonal Therapy

Rafael Contreras-Montoya,[#,1,2,3] Maria Sancho-Albero,[#,4,5,6,7] Catherine Adam,[#,1] Federico Movilla,[2,3] Mari C. Mañas-Torres,[8] Modesto T. López-López,[9,10] Angel Orte,[2,3] Victor Sebastian,[4,5,6,11,] Luis Álvarez de Cienfuegos[3,10,12,]* and Asier Unciti-Broceta[1,]**

[1] Cancer Research UK Scotland Centre, Institute of Genetics and Cancer, University of Edinburgh, Crewe Road South, Edinburgh EH4 2XU, UK.

[2] Nanoscopy-UGR Laboratory, Faculty of Pharmacy, University of Granada, E-18011, Granada, Spain.

[3] Unidad de Excelencia Química Aplicada a Biomedicina y Medioambiente (UEQ), Universidad de Granada, E-18071, Granada, Spain.

[4] Instituto de Nanociencia y Materiales de Aragon (INMA), CSIC− University of Zaragoza, E-50009 Zaragoza, Spain.

[5] Department of Chemical and Environmental Engineering Universidad de Zaragoza Campus Rio Ebro, E-50018 Zaragoza, Spain.

[6] Networking Research Center on Bioengineering, Biomaterials and Nanomedicine (CIBER-BBN), E-28029 Madrid, Spain.

[7] Instituto de Investigación Sanitaria (IIS) de Aragón, Avenida San Juan Bosco, 13, Zaragoza, 50009 Spain

[8] POLYMAT and Department of Applied Chemistry, Faculty of Chemistry, University of the Basque Country UPV/EHU, Paseo Manuel Lardizabal 3, Donostia-San Sebastián E-20018, Spain.

[9] Department of Applied Physics and Research Unit “Modeling Nature” (MNat), University of Granada, Avenida de Fuentenueva, E-18071 Granada, Spain.

[10] Instituto de Investigación Biosanitaria ibs.GRANADA, Av. de Madrid, 15, E-18016 Granada, Spain.

[11] Laboratorio de Microscopías Avanzadas, Universidad de Zaragoza, E-50018 Zaragoza, Spain.

[12] Department of Organic Chemistry, University of Granada, Avenida de Fuentenueva, E-18071 Granada, Spain.

[#] Equal contributions.

Table of contents:

1. Preparation and Characterization.. 2

2. Biological Methods. 6

3. In vivo experiments. 9

4. Supplementary Figures. 12

4. References. 22

1. Preparation and Characterization.

Pd nanosheets (PdNSh) synthesis.

PdNSh were synthesized by adapting previously reported protocols,[1] with modifications specifically designed to increase the yield of PdNSh. Briefly, a growth solution was prepared by dissolving 33 mg of Na_2PdCl_4, 90 mg of PVP (MW = 55,000), and 390 mg of KBr in 1200 µL of Milli-Q water. This mixture was combined with 12 mL of DMF to yield a homogeneous red solution, which was then transferred to a Teflon-lined, high-pressure stainless-steel reactor. To reduce the precursor and induce anisotropic growth of Pd nanocrystals, the reactor was pressurized with CO gas at 6 bar and heated in a water bath at 80 °C for 50 min under gentle magnetic stirring. The resulting dark blue colloid was recovered by centrifugation (10,000 rpm, 10 min) after adding acetone in a 1:3 volume ratio. Pd concentration was subsequently determined using MP-AES (Agilent 4100). The final PdNSh were redispersed in Milli-Q water (3.5 mg·mL^{-1}) and stored at 5 °C. TEM images showed that the PdNSh tend to stack during the drying process of the TEM grid preparation (Figure S1b). This effect enabled us to observe the ultrathin sheetlike morphology and made their direct thickness measurement easy.

MetallopetiGELs preparation.

To prepare 1 mL of gel, in a 2 mL test tube, 0.286 mL of 3.5 mg·mL^{-1} (concentration of Pd) PdNSh suspension were added to 0.714 mL of 7 mg·mL^{-1} alkaline (pH 9.5-10.0) solution of Fmoc-FF (Bachem) prepared as in procedure previously described.[2,3] The mixture was rapidly vortexed until a continuous and partially heterogeneous gel was obtained. The gel was then sonicated for 2 minutes in an ultrasonic bath to obtain a homogeneous gel. 1 mL of PBS buffer solution was then cautiously added on the gel surface and allowed to diffuse for 24 hours at room temperature. The supernatant was removed and the obtained gel stored at 4 ºC.

Rheology.

MetallopeptiGEL's mechanical robustness and self-healing behavior was analyzed with Haake Mars III controlled stress rheometer (Thermo Fisher Scientific, Waltham, MA, USA), provided with parallel plates of 35 mm of diameter. The gels were generated in 1 mL batches, as described above, and transferred into a disposable bottom plate of the

rheometer, on which a cylindrical hole of 2 mm depth and 35 mm diameter was previously carved. A Pasteur pipette was employed for gel transference until the well was filled.[2,3] The bottom plate/well was maintained at 37 °C throughout all experiments. The gel was allowed to stabilize in the plate at 37 ºC for 2 minutes before any rheological measurement. To study the mechanical behavior of the metallopeptiGELs, two different protocols were executed under oscillatory shear strain: i) the hydrogels were subjected to oscillatory shear strains of constant frequency (1 Hz) and stepwise increasing shear strain amplitude, γ_0, and ii) the samples were subjected to oscillatory shear strains of constant amplitude (γ_0 = 0.005) and stepwise increasing frequency. In both cases, the corresponding oscillatory shear stress was monitored and from these data the values of the viscoelastic moduli (storage modulus, *G'*, and loss modulus, *G"*) were obtained.

To evaluate the self-healing behavior, the viscoelastic moduli (storage modulus, *G'*, and loss modulus, *G"*) were monitored as function of time under an oscillatory shear strain of amplitude, γ_0 = 0.001 kept for 5 min for the 'gel regime', and γ_0 = 1.5 kept for 5 minutes for the 'sol regime', both at a frequency of 1 Hz. A total of 5 *gel-to-sol* cycles were studied for each replicate. For the last cycle, the gel's full self-healing was monitored at an oscillatory shear strain of amplitude, γ_0 = 0.001 for 30 minutes.

TEM.

Morphological characterization of the PdNSh and metallopeptiGELs was performed via Transmission Electron Microscopy (TEM) using a Tecnai FEI T20 microscope. The instrument operated at an accelerating voltage of 200 kV and was equipped with a LaB6 electron source and a SuperTwin objective lens, providing a point-to-point resolution of 2.4 Å. For sample preparation, 200-mesh copper (Cu) grids were utilized. Specifically, a 20 µL aliquot of the PdNSh colloid was drop-cast onto the grid. In the case of the hydrogel, a thin coating was applied to the grid surface to investigate its structural and morphological features.

Molecular Dynamics (MD) simulation.

The three-dimensional structure of Fmoc-FF was created using Avogadro software and the topology file was generated by the online server SwissParams with the CHARMM27 force field. The slab of 288 Pd atoms disposed in a FCC-111 plane fashion was generated with the Atomic Simulation Environment (ASE) on python, and its topology was coined

by hand taking an average of the σ and ε parameters previously reported for the Pd-C(sp^2) and Pd-O interaction.[4] To account for the rigidity of the nanonsheet and to avoid critical deviations during the NPT stabilization, all Pd atoms were frozen on their respective location.

Two different systems were designed to study independent phenomena: On one hand, one system presented a molecule-to-volume proportion that represented the macroscopic Fmoc-FF concentration employed during the experiment. This system allowed us to study the behavior of Fmoc-FF around the Pd nanosheet and give insights on the nature of the interaction between gelator and nanosheet. On the other hand, systems containing an increase concentration were also developed to study the potential role of gelator intermolecular interaction in the nanosheets assembly and relative orientation. Due to the necessity of representing a crowded inter-sheet space to account for the accommodation of multiple Fmoc-FF molecules, we decided to arbitrary increase the concentration until a satisfactory number of interactions could be observed.

The diluted systems were composed by 11 Fmoc-FF molecules and a Pd slab in a 10x10x10 nm cubic box, while the concentrated systems consisted of 55 Fmoc-FF molecules contained in the same volume. For all the cases, the slab was positioned at the center of the box. When double-slab system was studied, a second box with a centered slab was placed as an extension of the Z axis of the original box, and distances between slabs were selected to represent the same inter-nanosheet distance observed by TEM images (c.a. 3 nm). The water solvent was added to each box using the “solvate” command in GROMACS, employing an SPC/E model.

MD simulations were carried out using GROMACS 2023 software, with the CHARMM27 force field. This force field is not only versatile but also extensively utilized in diverse supramolecular simulations.[5]

After preparing these simulation boxes, energy minimization was performed using the steepest descent algorithm. To ensure a strictly minimized system the cut-off value for the maximum tolerable force was kept at < 800 kJ/mol, and the number of steps was increased up to 500,000. Afterwards, the energy-minimized structures were equilibrated at both constant volume and pressure. The LINCS algorithm was utilized to constrain only the bond lengths involving hydrogen. To achieve the desired temperature of 298 K, the modified Berendsen thermostat known as V-rescale was employed for 200 ps. To achieve a pressure of 1 bar, the Parrinello-Rahman barostat algorithm was utilized for 200 ps.

Compressibility was adjusted according to represent a diluted aqueous solution ($4.50 \cdot 10^{-5}$), and the Tau parameter was increased up to 2 to ensure slow and smooth coupling. Particle Mesh Ewald model was considered to account for long-range interactions, with 1 nm cut-off for short-range electrostatic and van der Waals interactions.

After the equilibration phases, the simulations were carried out for 100 ns.

Small Angle X-ray Scattering (SAXS).

SAXS experiments were conducted at the BL11 NCD-SWEET beamline of the ALBA Synchrotron Light Source (Barcelona, Spain), under project ID 20250340041.

Samples were measured using a custom-designed Kapton sample holder for liquids, providing a path length of 2 mm and all the measurements were carried out at room temperature. The X-ray beam was set to operate at an energy of 15 keV, and the distance between the sample and detector was set to 6700 mm. Two-dimensional patterns were captured using a Pilatus 1M detector (Dectris, Baden, Switzerland), capturing 5 consecutive images with a 5-second exposure time. The one-dimensional patterns were obtained through azimuthal integration using the pyFAI library,[6] and the intensity was expressed as a function of the scattering momentum transfer q (q=4*(π/λ)*$sin(\theta)$).

Data was modelled employing the SasView software.[7] To interpretate the results obtained from the nanosheets before and after the mixing with Fmoc-FF solution, a model based on a cylinder with a hard-sphere structure factor was employed.[8] The effective radius was computed from a sphere with an equivalent volume. To account for the polydispersion in the radius and the length, a Schultz distribution was introduced to the model.[9] To model the data from the Fmoc-FF solution, an axially elongated cylinder was best suited. For all cases, the Nelder-Mead simple fitting optimizer was employed.

2. Biological Methods.

Monitoring of fluorescence kinetics.

MetallopeptiGELs (15 µg Pd in 15 µL gel) or PdNSh (15 µg Pd in 15 µL water) were aliquoted into Eppendorf vials. **Poc-eNBD** (360 µL, 104 µM, 2% DMSO) in PBS or PBS+10% v/v fetal bovine serum (FBS) was cautiously added. Final concentration of Pd: 40 µg/mL. The tubes were incubated at 37 ºC for 1, 2, 4, 8, 24 and 36 h. At each time point, aliquots (160 µL) were transferred to a black 96 well-plate, and the conversion quantified by fluorescence measurements, compared to a calibration curve of **eNBD**+**Poc-eNBD** in PBS or PBS+serum. Fluorescence emission was detected using a Tecan infinite 200Pro plate reader (Ex/Em: 440/535 nm). The results are expressed as the mean of n=3 ±SD independent replicates prepared for each time point.

Prodrug activation in vitro.

MetallopeptiGELs (15 µg Pd in 15 µL gel) were aliquoted into Eppendorf vials. **DiPro-5FU** or **5-FU** (360 µL, 500 µM, 0.5 % DMSO) in Dulbecco's Modified Eagle Medium (DMEM) +10% FBS was carefully added over the gels and the tubes were incubated at 37 ºC for 24 h, then centrifuged. Final concentration of Pd: 40 µg/mL. Aliquots of the supernatant (20 µL) were analyzed by HPLC using a Waters AutoPurification system equipped with an XBridge C18 3.5 µm, 2.1x50 mm analytical column and diode array detector. Method: 5-50% MeCN / H20 (+ 0.1% v/v NH_3), 10 min, flow rate: 1 mL/min. HPLC traces were process at 254 nm using MassLynx software, and sample conversion quantified by calibration curve of **5-FU** + **diPro-5FU** in DMEM +10% FBS. The results are expressed as the mean of n=3 ±SD independent replicates.

Prodye activation: leached Palladium.

Aliquots of metallopeptiGELs (20 µg Pd in 20 µL gel) were added to transwell inserts (40 µm, PET, Sarstedt) and dialyzed in PBS at 37 °C for 48 h. Transwells were then moved to fresh wells containing DMEM+10% FBS. The metallopeptiGEL was incubated for 5 d at 37 °C. Pro-Res (5 µM) was added to wells and metallopeptiGEL incubated for 24 h at 37 °C and fluorescence emission was detected using a Tecan infinite 200Pro plate reader (Ex: 560 nm / Em: 590 nm).

Cell Culture.

BxPC-3 cells (ATCC CRL-1687) were grown in Roswell Park Memorial Institute (RPMI) media supplemented with 10% foetal bovine serum (FBS) and 2 mM L-glutamine, then incubated in a tissue culture incubator at 37 °C and 5% CO_2.

DiPro-5FU activation - endpoint live/dead assay (PrestoBlue).

Aliquots of metallopeptiGELs (20 µL / well) were added to sterile transwell inserts (40 µm, PET, Sarstedt) and dialyzed in sterile PBS at 37 °C for 48 h, prior to addition to cells. On the same day, BxPC-3 cells were seeded in a 24-well plate format at a density of 15,000 cells / well, then incubated for 48 h before treatment. Each well was then replaced with 700 µL of fresh media containing either 100 µM prodrug; 100 µM **5FU** with 0.1% DMSO; or 0.1% DMSO alone. Final concentration of Pd: 29 µg/mL. Transwell inserts containing the metallopeptiGELs were then transferred to the test-plate and incubated for 5 d. Inserts were then removed and each well was replaced with 500 µL fresh media, PrestoBlue cell viability reagent (10% v/v) was added to each well, and the plate was incubated for 2 h. Fluorescence emission was detected using a Tecan Spark 20M plate reader (Ex: 560 nm / Em: 590 nm). All conditions were normalized to DMSO cells (100%).

DiPro-5FU activation - timelapse apoptosis assay (Incucyte® S3 System).

Aliquots of metallopeptiGELs (ca. 20 mg / well) were added to transwell inserts (40 µm, PET, Sarstedt) and dialyzed in PBS at 37 °C for 24 h, prior to addition to cells. BxPC-3 cells were seeded in a 24-well plate format (80,000 cells / well) and incubated 24 h before treatment in simple media.

Each well was the replaced with 1 mL of fresh media containing either pro-Res (5 µM); Res (5 µM); diPro-5FU (100 µM); 5-FU (100 µM); or DMSO (0.1% v/v). Final concentration of Pd: 20 µg/mL. Transwell inserts containing the metallopeptiGELs were then transferred to the test-plate and incubated for 24 h. CellEvent™ Caspase-3/7 green detection agent (5 µM, Invitrogen™ C10723) was added and the plate transferred to the Incucyte® S3 system. The cells were imaged at 4 h intervals for 4 d in phase and green fluorescence channels. Images were all normalized to the same green / red intensity.

Palladium-leaching quantification.

Aliquots of cell media (1 mL) were removed after 5 d in cell culture – as above – from wells containing DMSO and DMSO+metallopeptiGELs. Samples were freeze-dried and resuspended in HNO_3 (200 µL, 70% v/v) and gently heated until all solids dissolved. The samples were then diluted into HNO_3 (1.8 mL, 10% v/v) and briefly centrifuged to separate white precipitate. Aliquots of the supernatant were removed and serially diluted into HNO_3 (10% v/v, 10-fold dilution), and water (10-fold dilution) and analysed as for Pd content as follows using a triple quadrupole inductively coupled plasma mass spectrometer (ICP-MS; type 8900, Agilent, USA). The ICP-MS was set up using the low matrix mode with the following key settings: Plasma, auxiliary and nebuliser gas flows of 15, 0.9 and 1.09 L / min respectively, RF power 1550W and the spray chamber cooled to 2 °C. Palladium was analysed in Helium mode where Helium gas is used in the collision cell was used to improve background noise, the Helium flow rate was 5 mL / min. A matrix matched external calibration curve for Palladium of the interference-free isotope 105 Palladium was performed over a range of 1-1000 µg / L at the start of the analysis. On-line addition and correction for drift and or matrix differences was carried out with an internal standard (200 µg / L 193 Iridium and the correction for the chemical blank were also applied. For the method validation, the limit of detection (LoD), was 0.002 µg/L. Samples were prepared for analysis by diluting 1:10 with deionised water (Type 1, Sartorius Arium Comfort II). The variation between analysis batches were determined by duplicate measurements of the samples.

3. In vivo experiments.

Animal and tumor model.

The procedures performed in this study were carried out under the Project License PI 43/24 approved by the Ethic Committee for Animal Experiments from the University of Zaragoza (*Comisión Ética Asesora para la Experimentación animal de la Universidad de Zaragoza*). Mice were fed ad libitum and their care and maintenance under specific pathogen-free conditions were performed accordingly with the Spanish Policy for Animal Protection RD53/2013, which meets the European Union Directive 2010/63 on the protection of animals destined to experimental and other scientific purposes.

For these experiments 6- to 8-week-old female BALB/c nu/nu mice (Envigo) were employed. All the animals were maintained under quarantine for 7 d upon arrival in the animal facilities and before starting the experiments. For the inoculation of the xenograft model, animals received a subcutaneous injection of 6 x 10^6 BxPC-3 cells in PBS mixed with Matrigel (media:Matrigel 1:1 *v:v*) in 200 mL as final volume. After tumor implantation mice were randomly divided in groups by tumor dimensions.

The procedures for the biodistribution and efficacy experiments are summarized in Figure S3 (including the mice groups, time-points, analysis and sampling).

In vivo biodistribution study.

Nine days after tumor implantation, 15 and 30 μL of metallopeptiGELs (corresponding to 15 and 30 μg of Pd, respectively) were administered intratumorally (IT). Then, mice were divided in 3 groups (8 animals per group): group 1, group 2 and group 3 were sacrificed after 1, 2 and 7 d after the metallopeptiGELs injection, respectively. Non-treated mice were used as control group. Animals were euthanized by CO_2 inhalation at the different time-points previously indicated.

To evaluate metal bioaccumulation, tumor, kidneys, liver, lungs, spleen and pancreas were collected from each animal (4 animals per group) and digested with aqua regia (from 1 to 6 mL based on the volume of the organ). After 5 d of digestion at room temperature, samples were diluted in 1/5 in miliQ water and the total amount of Pd present in each tissue was determined by ICP-OES (Perkin Elmer Elan DRC-e) in the Chemical Analysis Service of the University of Zaragoza.

For the histopathological analysis, tissues/organs were fixed in 4 % formaldehyde (Alfa Aesar) for 1 or 2 d (based on sample volume) using 2 animals per group. Tissues were then embedded in paraffin, and three micrometer sections were stained with hematoxylin and eosin (HE). The presence of the PdNSh of the metallopeptiGELs were observed under an inverted microscope (Olympus IX81). Also, to confirm the presence of the PdNSh of the metallopeptiGELs in the tumors (2 animals per group), they were frozen with isopentane cooled by liquid nitrogen and cryo-sectioned at 5 micrometres. Then, slices were assembled in a slide with FluoromentG and DAPI (ex/em 364/454) for visualization and observed under confocal microscopy (Spectral Confocal Microscope Zeiss LSM 880 with Airyscan). PdNSh were directly visualized by reflection of the incident light.

In vivo efficacy study.

Animals were divided in 4 groups (11 animals per group): group 1, untreated control; group 2, metallopeptiGELs only (30 µL, IT, once weekly); group 3, **diPro-5FU** only (150 mg/Kg, intraperitoneal (IP), 4 doses/cycle); and group 4, metallopeptiGELs (30 µL, IT, once weekly) and with **diPro-5FU** (150 mg/kg, IP, 4 doses/cycle). 5 animals of each group were sacrificed after 2 treatment cycles, and analyzed by immunohistochemistry, and 6 animals were sacrificed after receiving 3 treatment cycles to monitor tumor growth changes. Tumor size evolution was periodically evaluated using a calliper. To monitor mice health, animal weights were measured periodically (Figure S11).

Histopathological analysis. Animals were sacrificed by CO_2 inhalation. Caspase 3 immunostaining was used for the evaluation of the apoptosis in the tumor tissue. After antigen retrieval in TRIS-EDTA at pH 9 solution for 20 min at 97 °C (PT Link; Dako), endogenous peroxidase was blocked for 10 min with two washing steps with Dako washing buffer. Afterward, samples were incubated with the primary antibody anti-caspase 3 (Rabbit polyclonal, RyD Systems) diluted 1/125 during 30 min at room temperature, followed by the visualization system with the ImmPRESS HRP Horse Anti-Rabbit IgG Polymer Detection Kit and Peroxidase (VectorLabs, MP-7401). Finally, after three washing steps (Dako wash buffer, 5 min each) 3,3′-diaminobenzidine (DAB) was used for signal detection and hematoxylin for contrast.

Caspase 3 expression was semiquantified by counting positive and negative cells using QuPath software (version 6.0).[10] Parameters employed for cell detection and discrimination were a maximum background intensity of 2 with a radius of 2 and a

tolerable sigma of 0.3. Analyses were carried out including the cell nucleus in the detection and a cell expansion of 0.3. Due to the nature of the samples studied, the boundaries were selected as smooth, and to clearly separate touching cells we employed the Watershed algorithm, which allowed us to split touching objects by their shape. The scored compartment for each expression analysis was the cytoplasm, and a single threshold of 0.3 was employed to account for positive detection.

Statistical analysis of *in vivo* experiments.

The biological experiments were performed in triplicate. All the data are indicated as mean ± SD. Statistical analysis of the significant differences among the means were analyzed by one-way or two-way analysis of variance (ANOVA) for multiple comparisons by Dunnett's multiple comparisons test (GraphPad Prism Software). Statistically significant differences were expressed as follows: * $p<0.05$; ** $p<0.01$.

4. Supplementary Figures.

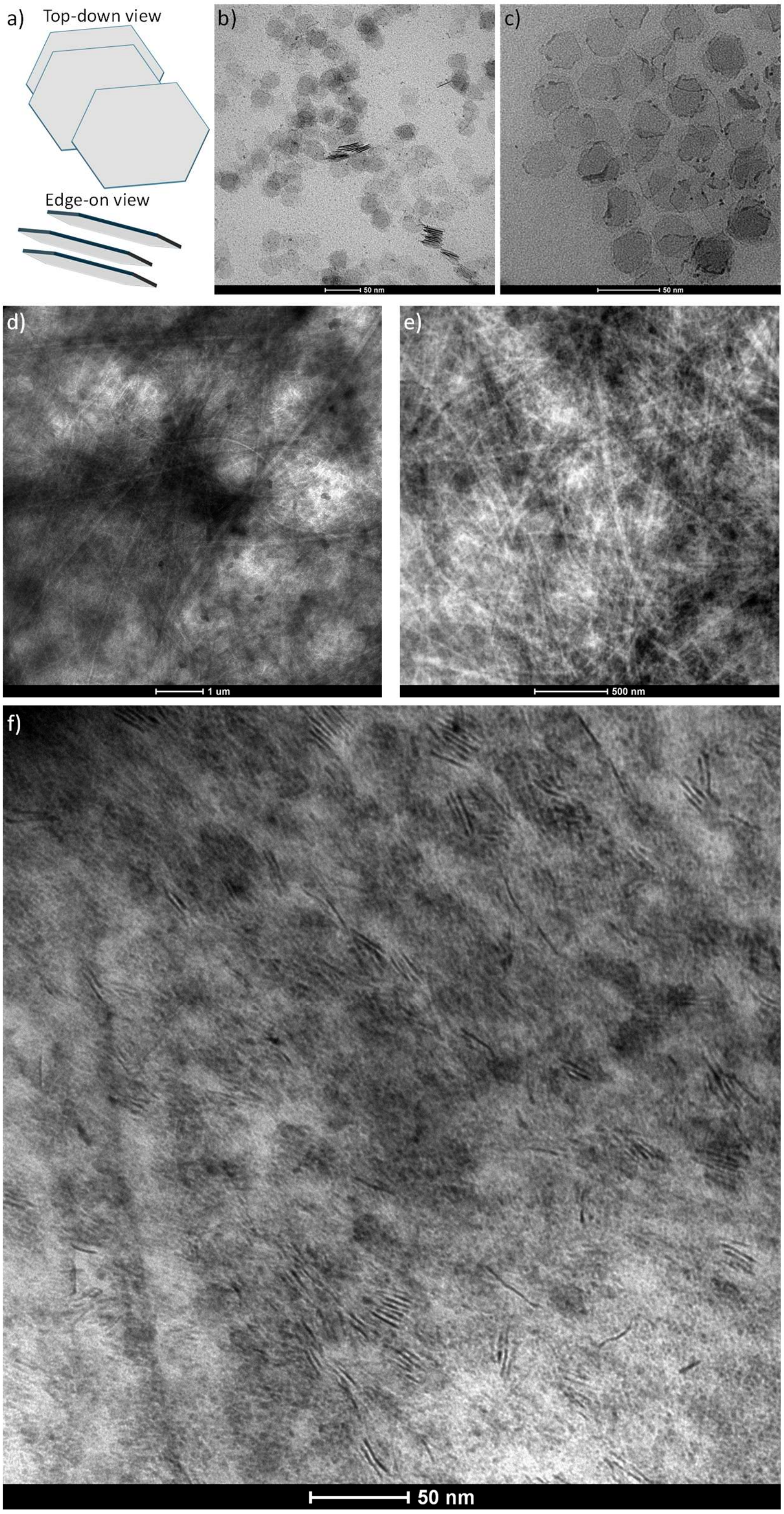

Figure S1. Structural and morphological analysis of the PdNSh and metallopeptiGELs. (a) Schematic illustration showing the top-down and edge-on views of the nanosheets. (b) Low-magnification TEM micrograph revealing a general overview of the nanosheets displaying representative orientations, including flat and edge-on profiles. (c) Higher-magnification TEM

image depicting the specific hexagonal geometry and surface morphology of individual sheets. (d–e) Low-magnification TEM micrographs providing an overview of the self-assembled fibrous network of the metallopeptiGELs. (f) TEM image illustrating the directional organization of PdNSh within the gel matrix. The nanosheets are predominantly observed in an edge-on orientation, appearing as thin, parallel nanostructures aligned along a preferential axis.

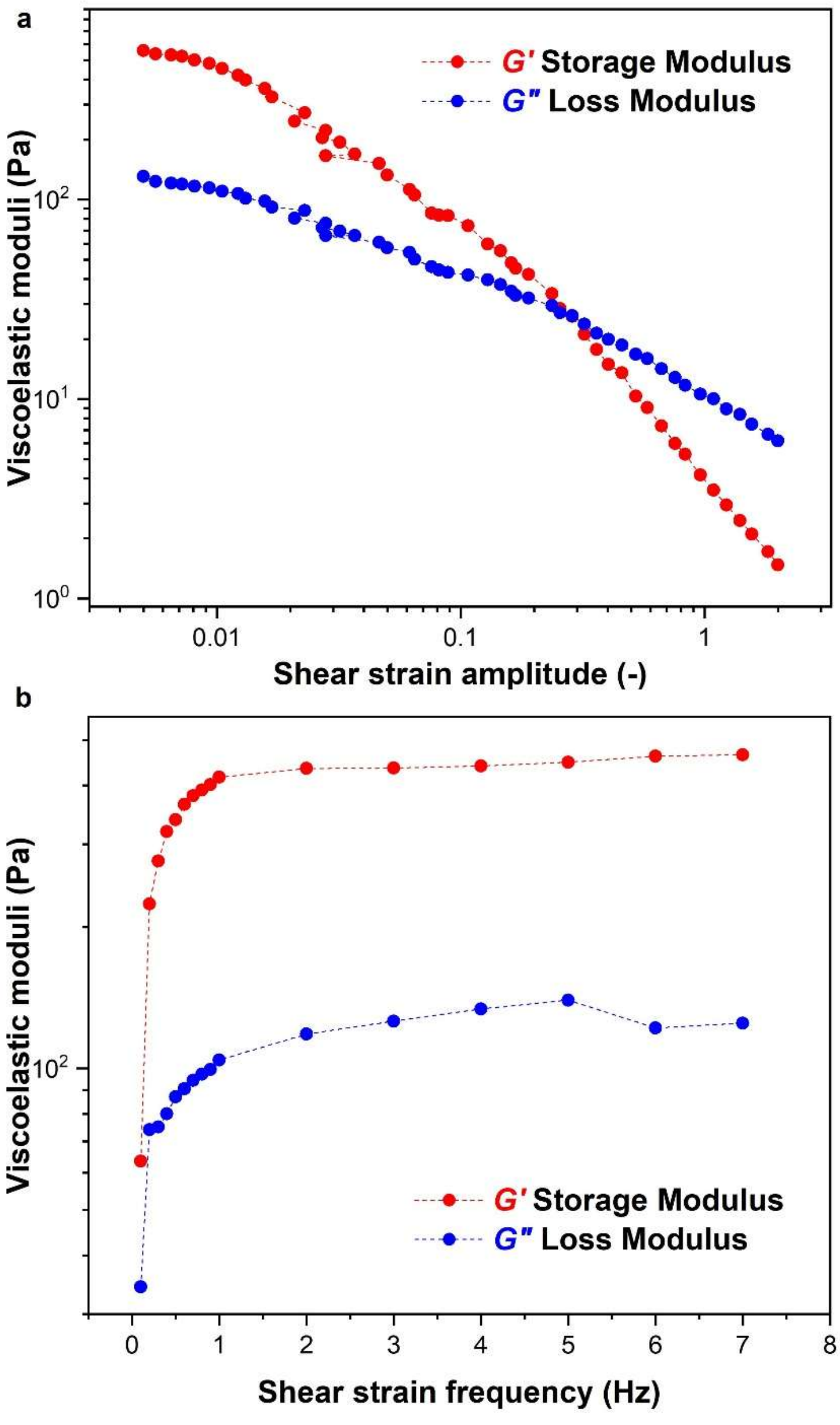

Figure S2. Rheological characterization of metallopeptiGELs. (a) Amplitude sweep. (b) Frequency sweep.

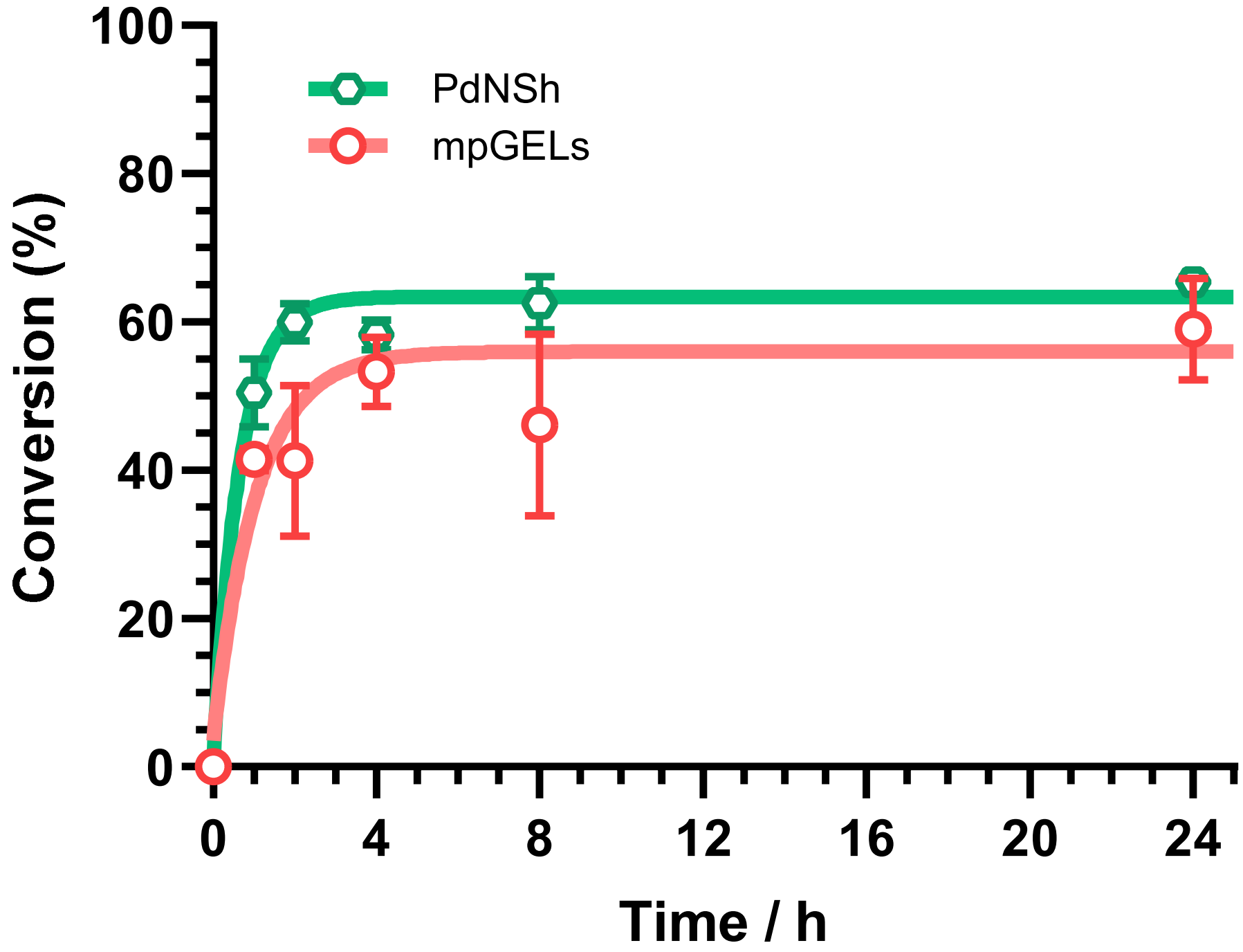

Figure S3. Palladium-mediated uncaging of Poc-eNBD (100 µM) into fluorescent eNBD catalyzed by metallopeptiGELs or PdNSh (40 µg/mL in Pd) in PBS at 37 °C.

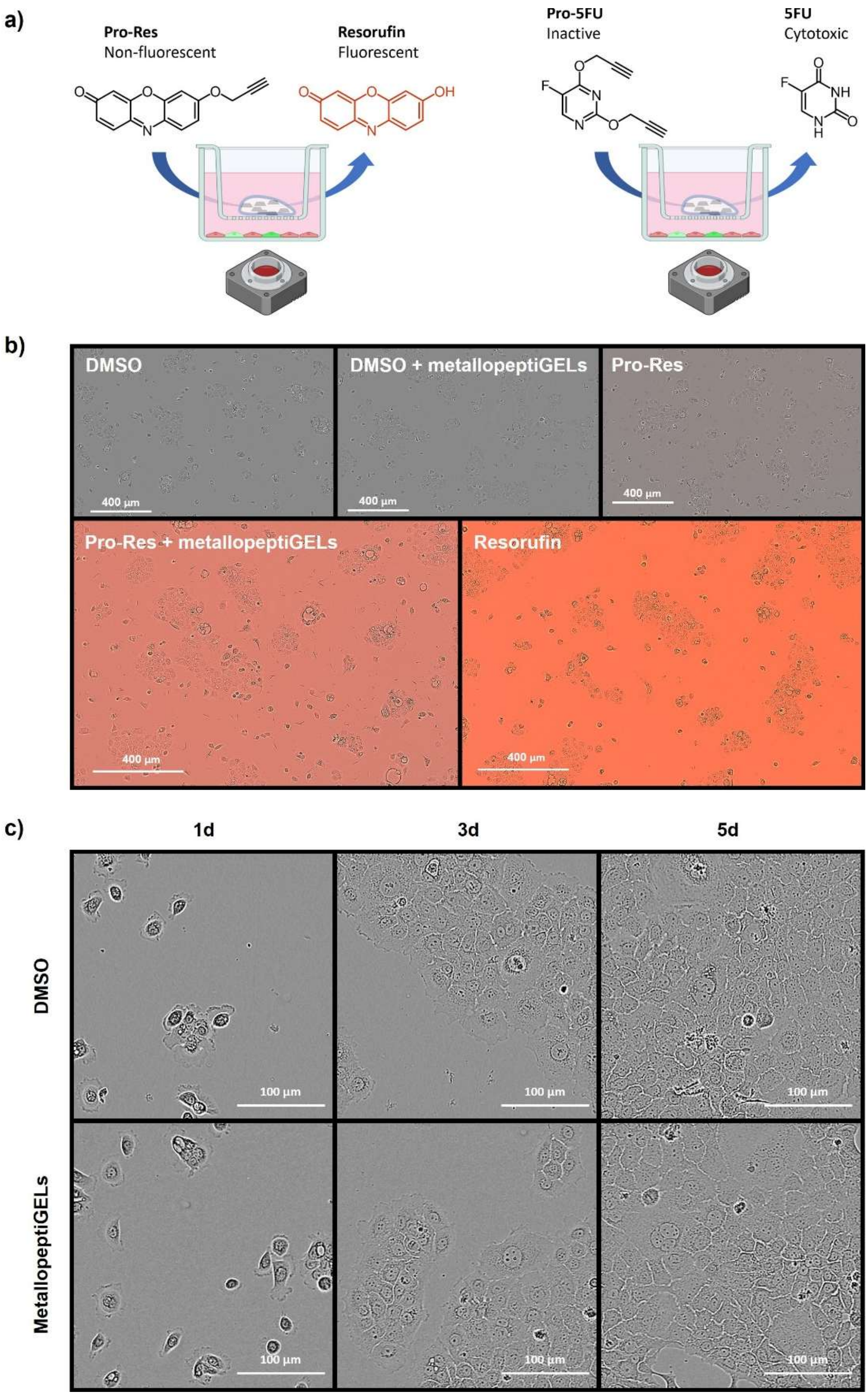

Figure S4. a) Experimental design. BxPC-3 cells were grown in a microplate, with metallopeptiGELs supported in a transwell insert, which allows free movement of small molecules across a semipermeable barrier. Cells were imaged at regular intervals with the IncuCyte S3 system in phase and fluorescent channels to simultaneously monitor cell growth and fluorescent dye release. b) Merged phase contrast and red-fluorescent images of cells after 4h incubation with DMSO, metallopeptiGELs (20 µg/mL in Pd), **Pro-Res** (100 µM), **Pro-Res** +

metallopeptiGELs or **Resorufin** (100 µM). c) Representative phase contrast images of metallopeptiGELs in culture with BxPC-3 pancreatic cells at day 1, 3 and 5. BxPC-3 cells were treated with DMSO (0.1% v/v); with and without metallopeptiGELs (20 µg/mL in Pd).

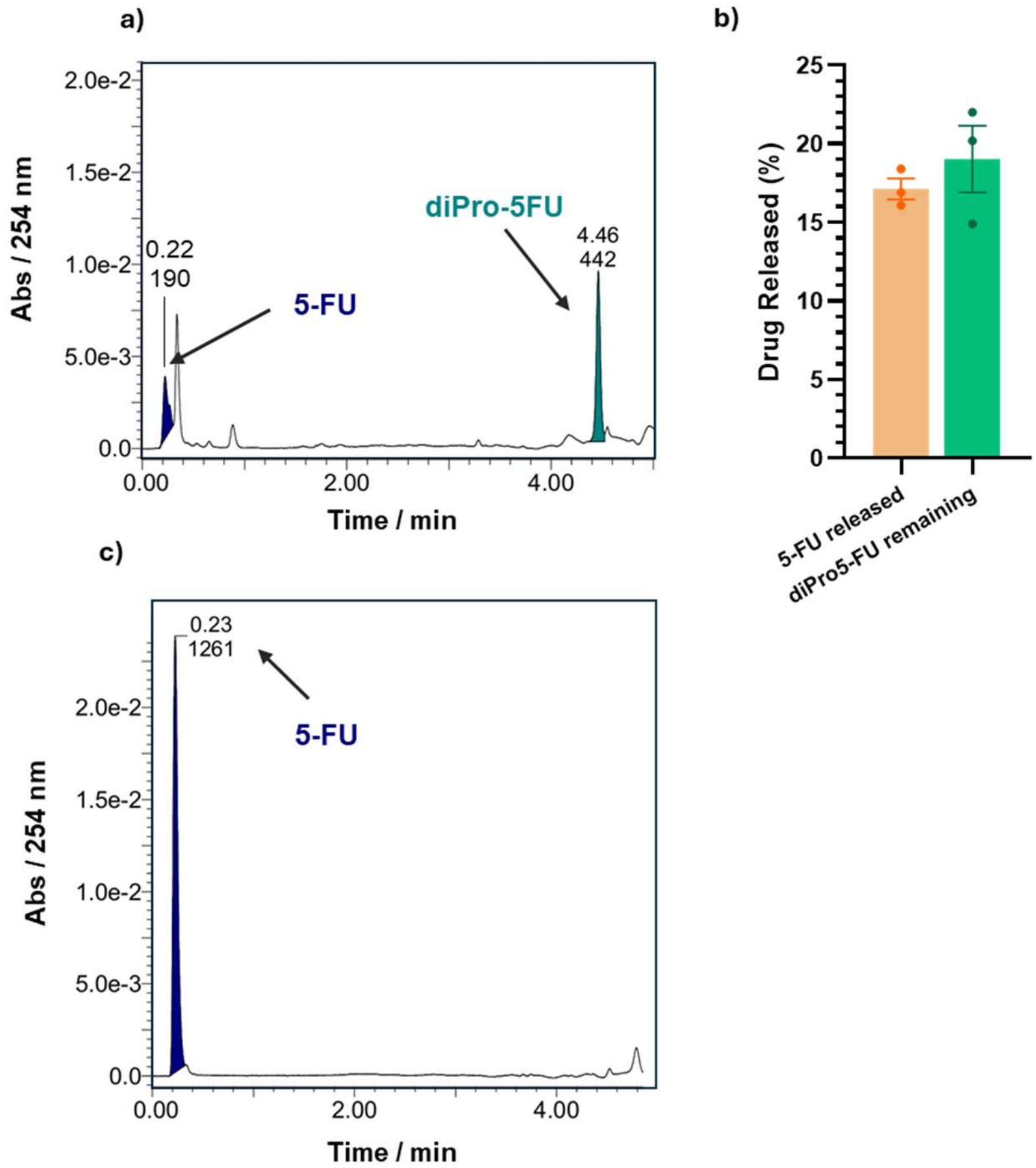

Figure S5. Prodrug activation study. **a**) LC-MS chromatogram of media after 1 d incubation activation of **diPro-5FU** (500 µM) by metallopeptiGELs (40 µg/mL in Pd) for 4 h. **b**) Quantification of **5FU** and **diPro-5FU** present in the media after 1 d incubation of **diPro-5FU** at 37 ºC with metallopeptiGELS. **c)** LC-MS chromatogram of control experiment: media after incubation of 5FU (500 µM) with metallopeptiGELs (40 µg/mL in Pd). Note that a high concentration of **diPro-5FU** (5-fold higher in other experiments) was required to be able to detect drug and prodrug by LC-MS.

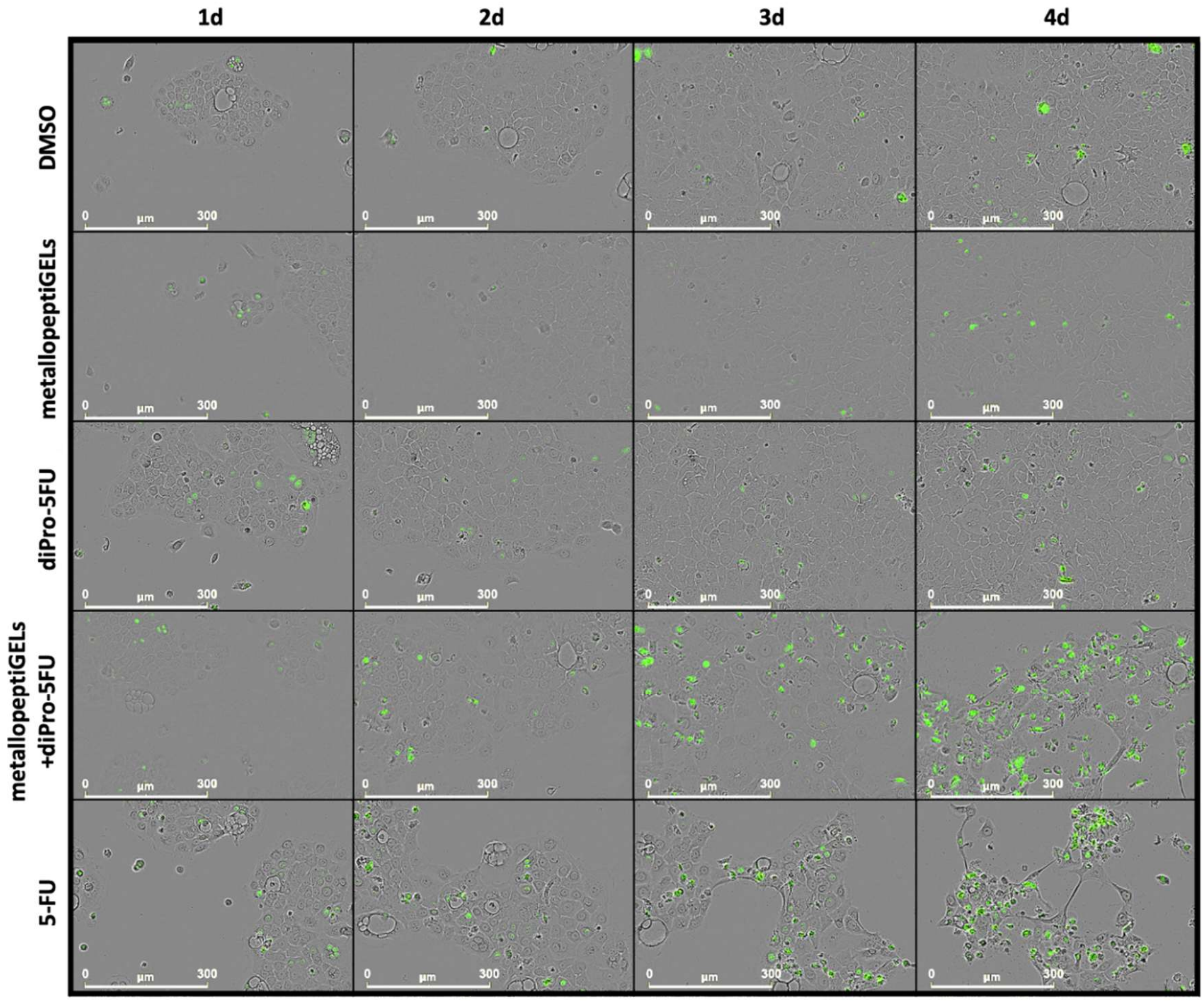

Figure S6. Apoptotic assay. Representative images of the metallopeptiGEL-mediated uncaging of **diPro-5FU** in culture with BxPC-3 pancreatic cancer cells after treatment for 1, 2, 3 and 4 d. Treatments: DMSO (0.1% v/v); **diPro-5FU** (100 µM) with and without metallopeptiGELs (20 µg/mL in Pd); and **5FU** (+ve control, 100 µM). Caspase-3/7 green detection reagent (CellEvent™ Caspase-3/7) was added 1 d after addition of the treatments and cancer cells were imaged with phase contrast and green-fluorescent channels using an Incucyte® S3 system every 4 h. See Supplementary Movie S2 for a timelapse of all timepoints.

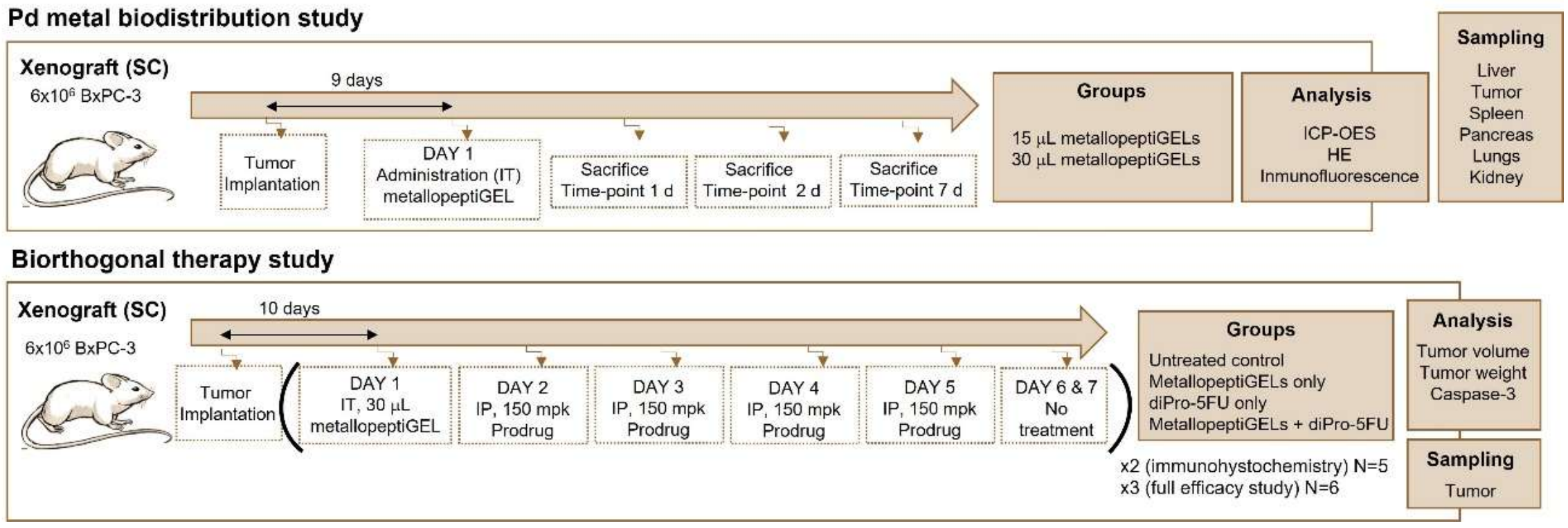

Figure S7. Experimental procedures followed in the human BcPC-3 xenograft study in mice. (a) Design for the Pd metal biodistribution study. (b) Description of the biorthogonal therapy cycles.

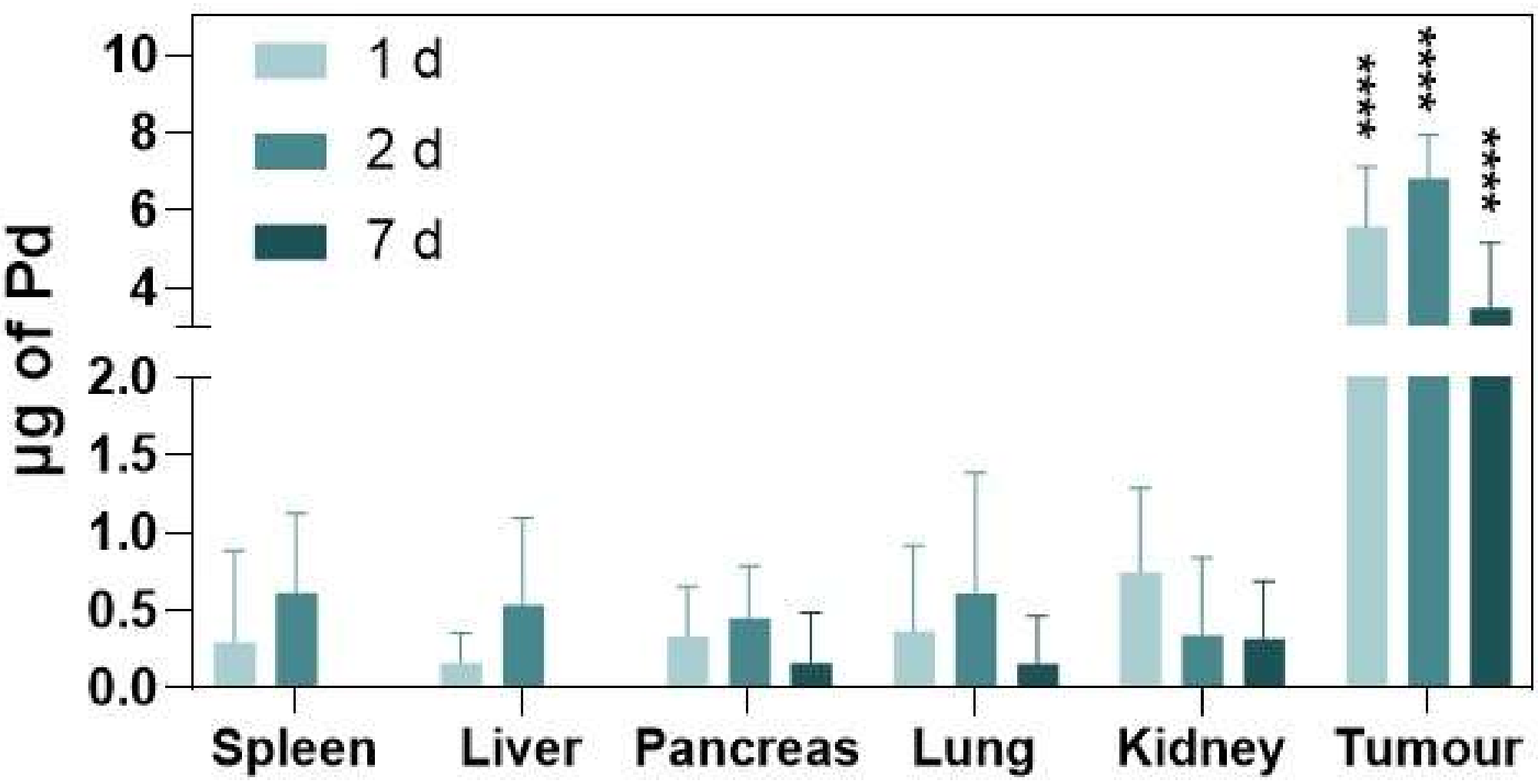

Figure S8. Pd biodistribution after intratumoral injection with 15 μL of metallopeptiGELs. Analysis performed by ICP-OES.

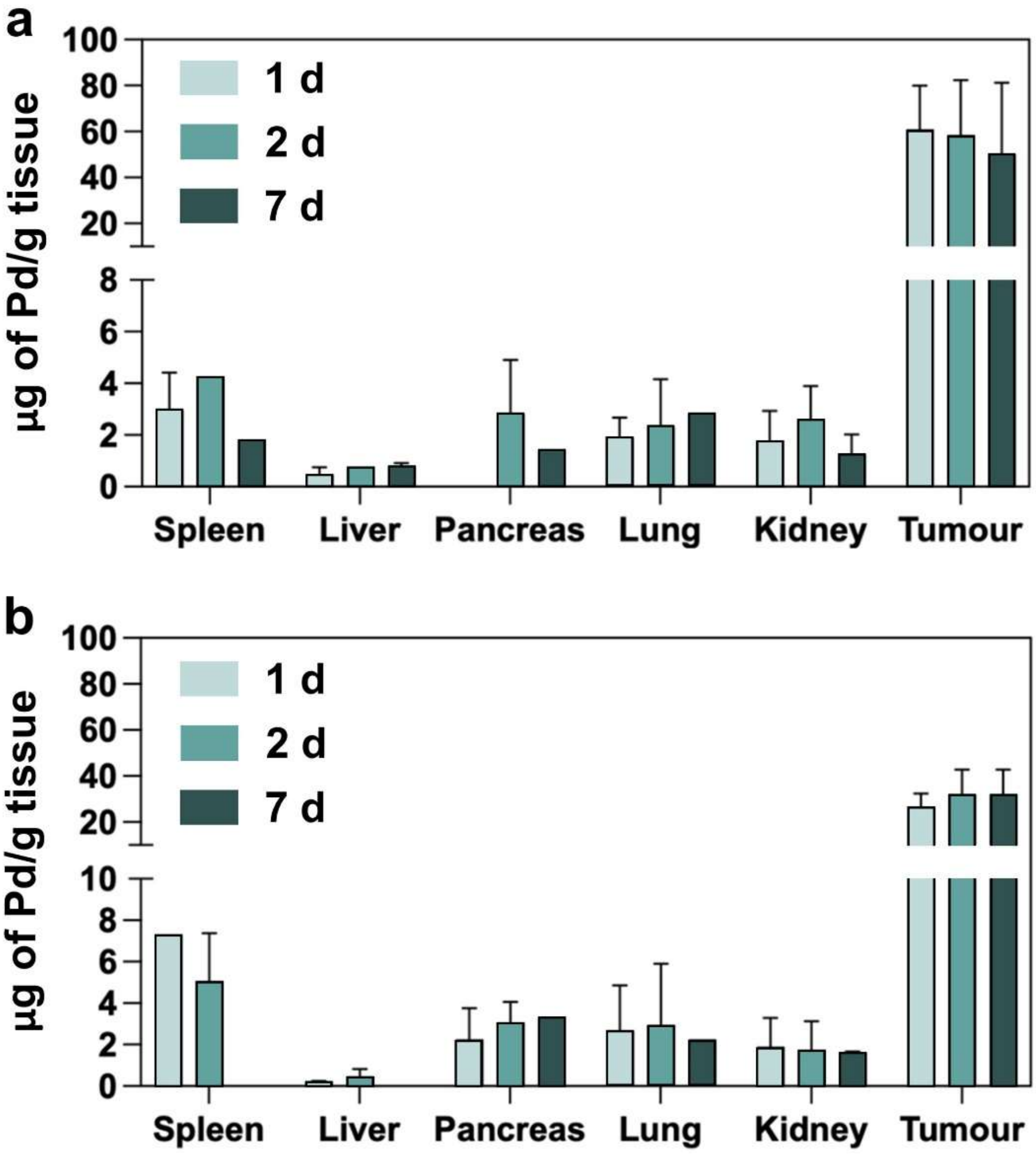

Figure S9. Quantification of μg of Pd normalized to *ex vivo* organ mean weight of mice treated with (a) 30 μL or (b) 15 μL of metallopeptiGELs.

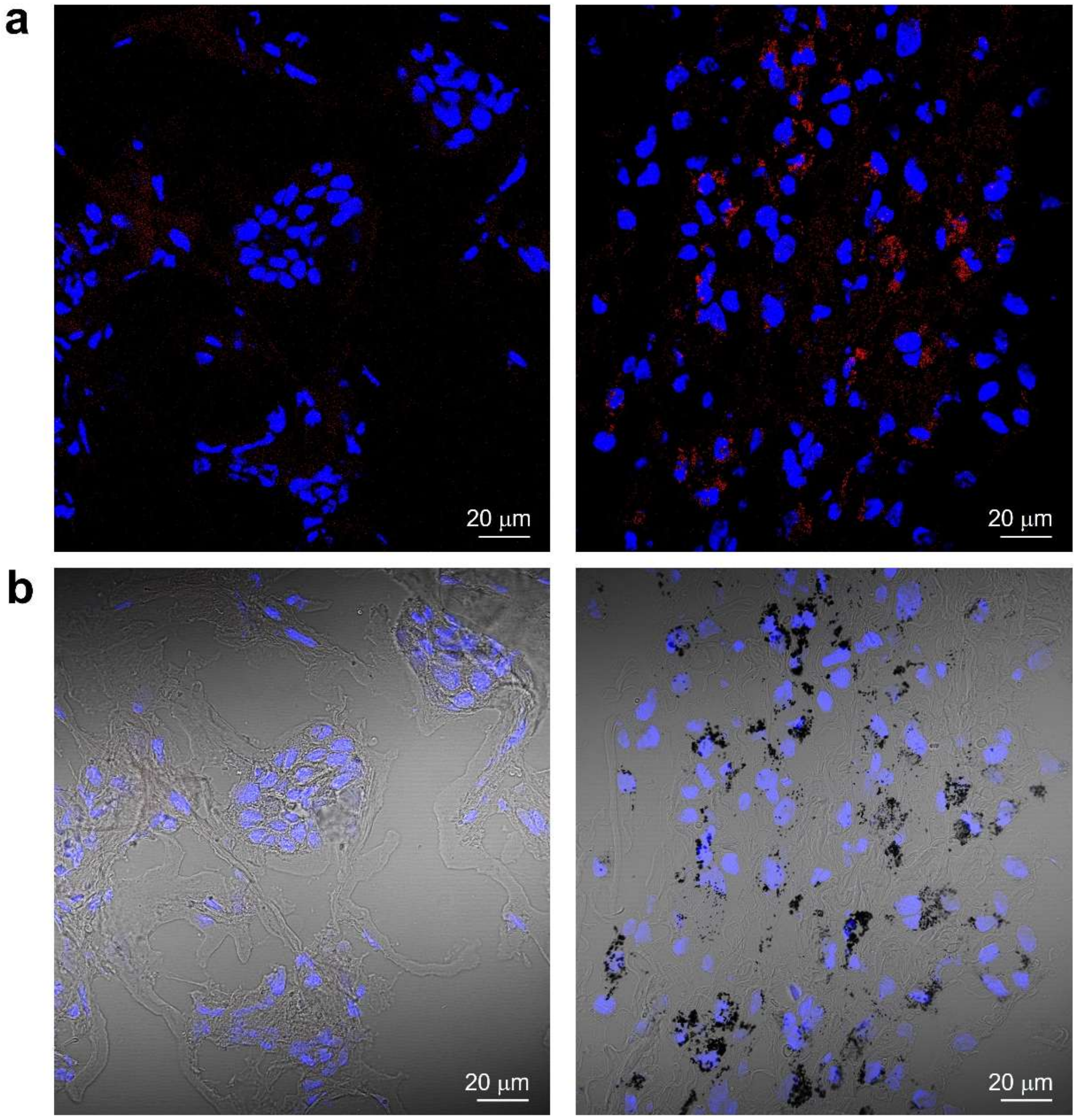

Figure S10. Confocal microscopy images of sliced tumors of untreated mice (left) and mice treated with 30 μL metallopeptiGELs (right): (a) merged fluorescent channels; (b) phase contrast images merged with DAPI channel. The nuclei of the cancer cells were marked with DAPI and appear in blue, while the metallic Pd nanoparticles are observed by reflection of the incident light as red (a, fluorescent images) or black aggregates (b, phase contrast).

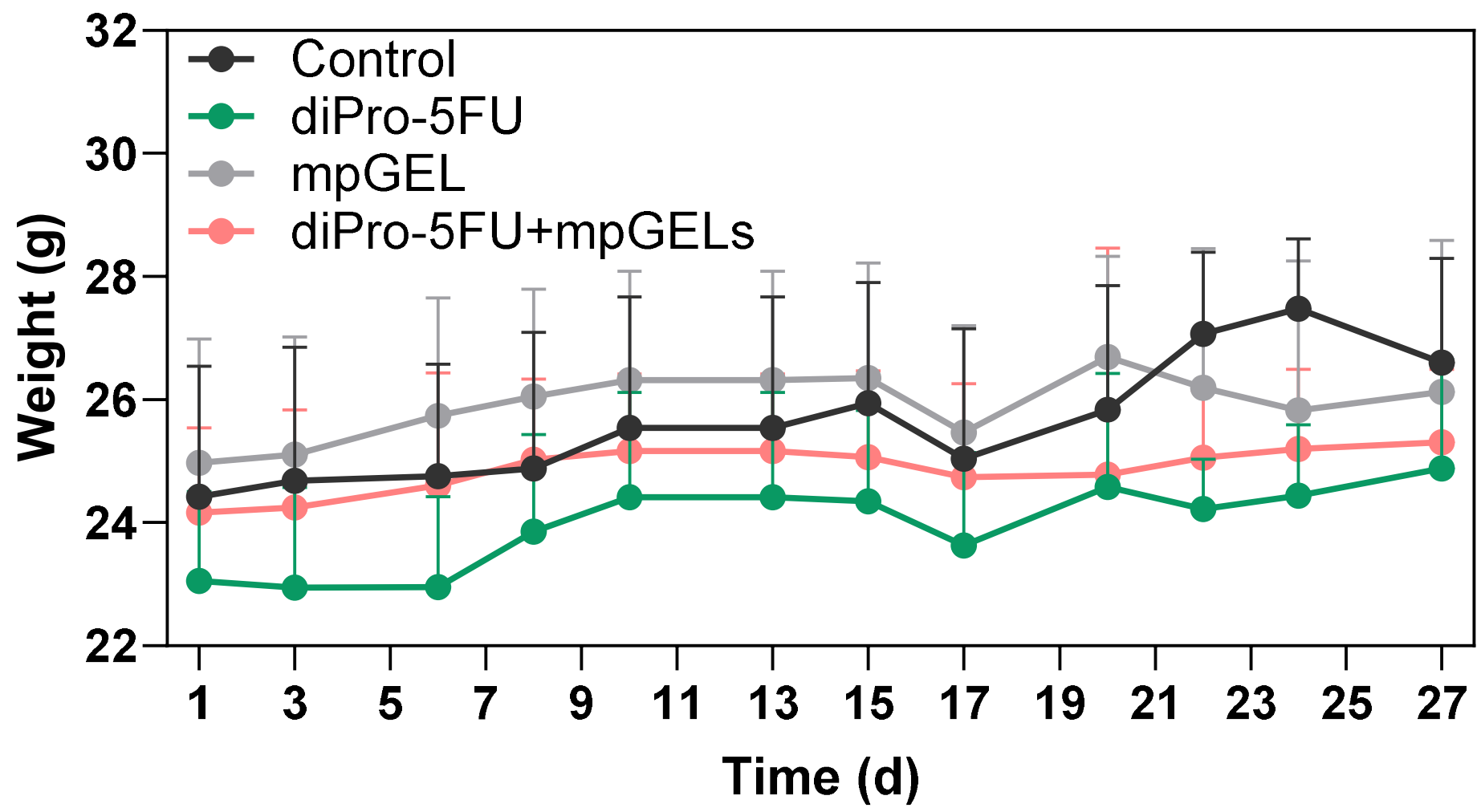

Figure S11. Evolution of animal weights during the *in vivo* therapeutic experiments. Note that almost half of the mice were sacrificed on day 17, artificially impacting the expected progression of animal weights in all groups.

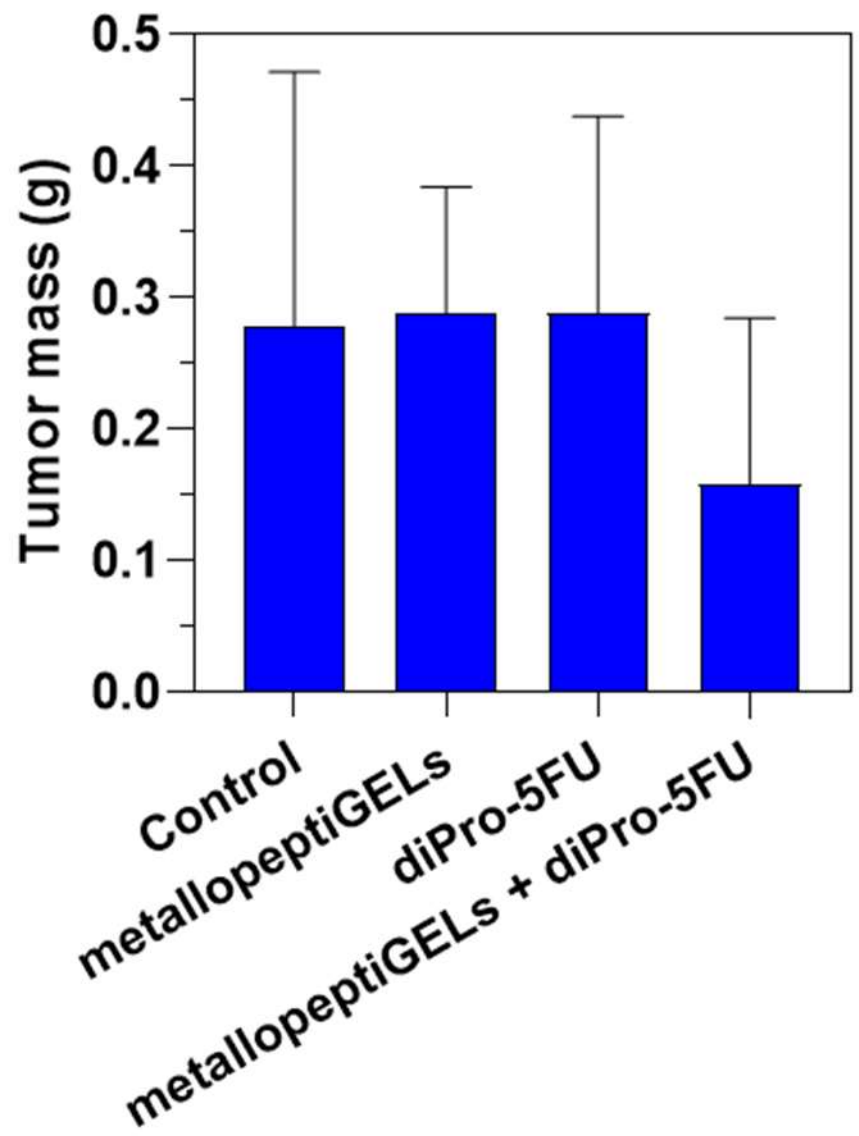

Figure S12. Tumor weights of mice sacrificed at the end of the experiment.

4. References.

(1) Pérez-López, A. M.; Rubio-Ruiz, B.; Valero, T.; Contreras-Montoya, R.; Álvarez de Cienfuegos, L.; Sebastián, V.; Santamaría, J.; Unciti-Broceta, A. Bioorthogonal Uncaging of Cytotoxic Paclitaxel through Pd Nanosheet–Hydrogel Frameworks. *J. Med. Chem.* **2020**, *63* (17), 9650–9659. https://doi.org/10.1021/acs.jmedchem.0c00781.

(2) Contreras-Montoya, R.; B. Bonhome-Espinosa, A.; Orte, A.; Miguel, D.; M. Delgado-López, J.; G. Duran, J. D.; M. Cuerva, J.; T. Lopez-Lopez, M.; Cienfuegos, L. Á. de. Iron Nanoparticles-Based Supramolecular Hydrogels to Originate Anisotropic Hybrid Materials with Enhanced Mechanical Strength. *Mater. Chem. Front.* **2018**, *2* (4), 686–699. https://doi.org/10.1039/C7QM00573C.

(3) Contreras-Montoya, R.; Escolano, G.; Roy, S.; Lopez-Lopez, M. T.; Delgado-López, J. M.; Cuerva, J. M.; Díaz-Mochón, J. J.; Ashkenasy, N.; Gavira, J. A.; Álvarez de Cienfuegos, L. Catalytic and Electron Conducting Carbon Nanotube–Reinforced Lysozyme Crystals. *Adv. Funct. Mater.* **2019**, *29* (5), 1807351. https://doi.org/10.1002/adfm.201807351.

(4) Hughes, Z. E.; Walsh, T. R. Distinct Differences in Peptide Adsorption on Palladium and Gold: Introducing a Polarizable Model for Pd(111). *J. Phys. Chem. C* **2018**, *122* (34), 19625–19638. https://doi.org/10.1021/acs.jpcc.8b05860.

(5) M. Frederix, P. W. J.; Patmanidis, I.; J. Marrink, S. Molecular Simulations of Self-Assembling Bio-Inspired Supramolecular Systems and Their Connection to Experiments. *Chem. Soc. Rev.* **2018**, *47* (10), 3470–3489. https://doi.org/10.1039/C8CS00040A.

(6) Ashiotis, G.; Deschildre, A.; Nawaz, Z.; Wright, J. P.; Karkoulis, D.; Picca, F. E.; Kieffer, J. The Fast Azimuthal Integration Python Library: pyFAI. *J. Appl. Crystallogr.* **2015**, *48* (2), 510–519. https://doi.org/10.1107/S1600576715004306.

(7) Doucet, M.; Cho, J. H.; Alina, G.; Bakker, J.; Bouwman, W.; Butler, P.; Campbell, K.; Gonzales, M.; Heenan, R.; Jackson, A. SasView Version 4.1. Zenodo. *SasView.* **2017**.

(8) Pedersen, J. S. Analysis of Small-Angle Scattering Data from Colloids and Polymer Solutions: Modeling and Least-Squares Fitting. *Adv. Colloid Interface Sci.* **1997**, *70*, 171–210. https://doi.org/10.1016/S0001-8686(97)00312-6.

(9) Kotlarchyk, M.; Stephens, R. B.; Huang, J. S. Study of Schultz Distribution to Model Polydispersity of Microemulsion Droplets. *J. Phys. Chem.* **1988**, *92* (6), 1533–1538. https://doi.org/10.1021/j100317a032.

(10) Bankhead, P.; Loughrey, M. B.; Fernández, J. A.; Dombrowski, Y.; McArt, D. G.; Dunne, P. D.; McQuaid, S.; Gray, R. T.; Murray, L. J.; Coleman, H. G.; James, J. A.; Salto-Tellez, M.; Hamilton, P. W. QuPath: Open Source Software for Digital Pathology Image Analysis. *Sci. Rep.* **2017**, *7* (1), 16878. https://doi.org/10.1038/s41598-017-17204-5.
